# MANGF: a reference library of DNA barcodes for Mantodea from French Guiana (Insecta, Dictyoptera)

**DOI:** 10.3897/BDJ.13.e149486

**Published:** 2025-04-09

**Authors:** Nicolas Moulin

**Affiliations:** 1 Institut Systématique, Evolution, Biodiversité, Paris, France Institut Systématique, Evolution, Biodiversité Paris France; 2 Entreprise Nicolas Moulin Entomologiste, Montérolier, France Entreprise Nicolas Moulin Entomologiste Montérolier France

**Keywords:** DNA barcoding, COI, molecular identification, cryptic diversity, Mantodea, predatory insects, ecological indicators

## Abstract

**Background:**

Mantodea plays a special role in the food chain as a group charismatic generalist predators. They regulate invertebrate populations while themselves being prey for many larger animals such as reptiles and birds. The present study focuses on Fench Guiana where about 78 species are known within eight families. This diversity represents a challenge for specimen identification.

**New information:**

The MANGF project aims at developing a DNA metabarcoding approach to facilitate and enhance the monitoring of mantises as indicators in ecological studies. As a first step towards that goal, we assembled a library of DNA barcodes using the standard genetic marker for animals, i.e. a portion of the COI mitochondrial gene. In the present contribution, we release a library including 425 records representing 68 species in eight different families. Species were identified by expert taxonomists and each record is linked to a voucher specimen to enable future morphological examination. We also highlight and briefly discuss cases of low interspecific divergences, as well as cases of high intraspecific divergences that might represent cases of overlooked or cryptic diversity.

## Introduction

The Mantodea is a charismatic predatory insect order with an estimated number of probably more than 2,500 species world-wide (Liu et al. 2023, Ma et al. 2023, Govorov et al. 2024, Otte et al. 2025). The raptorial legs, wings, morphology and genitalia characterise this order (Brannoch et al. 2017). However, evolutionary convergence can lead to unrelated species being similar (Christin et al. 2010, e.g. *Deroplatys* genus (Zhang and Price 2024) and *Brancsikia* genus (Saussure and Zehntner 1895, Roy and Schütte 2016) and potentially lead to misidentifcation (Moulin and Roy 2020). Examination of male genitalia through dissection is often required to confirm species identity, but this is not an easy process requiring laborious preparation and expertise. In addition to these challenges, species descriptions are often based on one sex only (more commonly males), making male/female association difficult, especially when sexually dimorphic species are involved. For several years, the use of DNA barcodes has modernised biodiversity research (Hebert et al. 2003, Hebert et al. 2003, Janzen et al. 2009, Maggia et al. 2021). DNA barcode libraries are being developed at a steady pace, combining genetic data (usually the sequences of the genetic marker used as the standard DNA barcode in animals: a 658 bp fragment of the mtDNA COI gene, although additional markers are sometimes used to completement it), taxonomic information and specimen data (collecting information, voucher repository, images). A global online database, the Barcode of Life Datasystems (BOLD), serves as the central repository for these libraries (www.boldsystems.org) and combines classical database features with a workbench facilitating data analyses and data sharing (Ratnasingham and Hebert 2007). Even though various effects may limit the efficiency of a successful species identification, for example, human errors (e.g. Carolan et al. (2012), Cheng et al. (2023)), geographical scale effect (e.g. Gaytán et al. (2020)), recent or ongoing hybridisation events (e.g. Rougerie et al. (2012), Rougerie et al. (2015), Gemmell et al. (2014), Dupont et al. (2016), Mutanen et al. (2016)), mitochondrial DNA-like sequences in the nucleus (numts) (e.g. Song et al. (2008), Moulton et al. (2010), Leite (2012), Jordal and Kambestad (2013), Ožana et al. (2022), Hebert et al. (2023), Nabholz (2023)) or effects of *Wolbachia* infections (e.g. Bilousov et al. (2011), Smith et al. (2012), Klopfstein et al. (2016), Kolasa et al. (2018), Kajtoch et al. (2019), Jiménez‐Florido et al. (2024)), DNA barcoding has become the method of choice in terms of modern molecular species identification, including the identification of single specimens as well as metabarcoding of bulk samples (e.g. Brandon-Mong et al. (2015), Moulin (2020), Decaëns et al. (2021), Sire et al. (2023), Rougerie et al. (2024)). In recent years, various barcode libraries for some insect taxa of French Guiana were established, for example, Diptera (Talaga et al. 2017, Boucher and Savage 2022) and Hymenoptera (Rongier et al. 2023); but only the first steps for Mantodea. Work has recently begun on this order, for example, in the Central African Republic (Moulin et al. 2017), in French Guiana (Moulin and Roy 2020) and in Cameroon (Govorov et al. 2024).

Mantids can be found in all habitats, rarely further north or south of 45° latitudes (Ma et al. 2023). Most species are distributed in tropical and subtropical habitats across the globe. In South America, mantids are very common and very diverse. In the Amazon Basin, French Guiana covers an area of 85,000 km², mostly covered by rainforests (90%, Rongier et al. (2023)), followed by coastal savannah, Inselberg and urban development (Young 2009). French Guiana is in the oldest and most homogeneous part of the Guiana Shield in South America (Guitet et al. 2015). However, it does not belong to a biodiversity hotspot as defined by Myers et al. (2000) because it is not an area with a strong level of endemism nor one that encompasses severely threatened ecosystems. Nevertheless, due to its high preserved forest coverage rate, it is recognised as part of the 24 wilderness areas in the world as defined byMittermeier et al. (2003). Despite a backdrop of a shortage of taxonomists (Engel et al. 2021), mantids from French Guiana are very well studied, due to combined efforts of few professional taxonomists and the large amateur community involved in collecting material (Touroult and Stéphane 2014, François and Roy 2015, Roy 2002a, Roy 2002b, Roy 2002c, Roy 2003, Roy 2004a, Roy 2004b, Roy 2005, Roy 2006, Roy 2010, Roy 2011, Roy 2012, Roy 2015, [Bibr B12486617][Bibr B12486572], Moulin and Roy 2020, Roy 2010, Roy 2011, Roy 2012, Roy 2015, Roy 2019, Moulin 2023, Moulin and Schwarz 2023).

In this study, we present a comprehensive DNA barcode library for the molecular identification of French Guiana Mantodea. This barcode library included 68 species of Mantodea, representing 42 genera in eight families. A total number of 425 DNA barcodes were examined in detail. We expect that this library development in the near future will further contribute to the assembly of a DNA barcode library for Amazonian Mantodea.

## General description

### Purpose

This library aims to provide an authoritative reference library for the DNA-based species identification of Mantodea from French Guiana, to facilitate the use of DNA metabarcoding in biodiversity monitoring networks focusing of these predatory insects. It is also expected to develop the use of DNA barcodes by the community of dictyopterists, in combination with characters from the morphology, ecology and biogeography of species, to address taxonomic questions.

### Additional information

The MANGF library uses the standard DNA barcode for animals, i.e. a 658 bp fragment of the COI mitochondrial gene.

Species identifications were provided by two expert taxonomists for these groups, Roger Roy and the author. All records were initially identified, based on morphological examination and vouchers are preserved in the collections of the Muséum national d’Histoire naturelle (MNHN), of Nicolas Hausherr and of the author as references for these records. Any future change in the taxonomy/nomenclature of these insects will be reported in the MANGF library, after authoritative validation by the taxonomists.

## Project description

### Title

MANGF: Mantodea DNA sequences from Research Collection of Nicolas Moulin and specimens from the MNHN and others sampling in French Guiana.

### Personnel

Nicolas Moulin (independent researcher, honorary attached to MNHN, Paris (ISYEB).

### Study area description

French Guiana (100% of the samples).

### Funding

This project was partly supported by the MNHN, Paris (PatriNat, centre of expertise and data on natural heritage).

## Sampling methods

### Study extent

The MANGF library focuses on Mantodea from French Guiana.

### Sampling description

Tissue samples for DNA extraction were collected mostly from dry collection specimens; only a limited number of samples were preserved in 95% ethanol. All specimens were photographed and specimen data were compiled in excel spreadsheets for submission to BOLD.

Most specimens were sampled by the author in his own research collection and in the collection of the MNHN. Nicolas Hausherr sampled specimens in his own reference collection.

### Quality control

All tissue samples were assembled in 96-well plates in which one well (location H12) was left empty to serve as a negative control. After sequencing and uploading of the sequences into BOLD, DNA barcodes were compared through classical analyses of genetic distances (BLAST hits, NJ trees) to conspecific records, when existing, in other accessible DNA barcoding projects/campaigns. Discordances between DNA results and taxonomy derived from morphology (DNA barcodes shared by distinct species, deep intraspecific splits (> 2%)) led to re-examination of the specimens; extensive research has been undertaken to resolve these problems by uncovering possible cases of misidentification or cross-contamination.

### Step description

The construction of the MANGF library can be divided into two steps:

**1. Specimen sampling and data compilation**:

- tissue sampling. Using flame-decontaminated forceps, we usually pulled part of the mesothoracic leg (tarsus, tibia, femur, depending on the size of the specimen) from each one sampled. For the smallest specimens, such as very young nymphs, we used part of the body or the whole specimen (in this case, we did not preserve any voucher specimen, only if these are nymphs that have just been born).
- photography. Each specimen was photographed individually along with a scale.
- data compilation. We used standard BOLD spreadsheets to compile:
- voucher information: SampleID (a unique BOLD identifier for the specimen; also added on a label pinned with the voucher specimen) and institution storing.
- taxonomy data: higher level taxonomy; species identification; identifier, including contact information.
- specimen details: sex (when available); reproduction mode; life stage.
- collection data: collectors; date of collection; country; administrative region (as sector); exact site; latitude, longitude and elevation (when available).
- upload to BOLD. Following the standard BOLD procedure for DNA barcode library construction, a dedicated project was created in BOLD. This project (code MANGF, publicly accessible) hosts records for all the samples processed (including failures), whereas the actual MANGF library (dataset DS-MANGF, see the *Data resources* section below) only includes records successfully sequenced and subsequently validated by taxonomists.

**2. Sequencing of DNA barcodes**: The Canadian Centre for DNA Barcoding (CCDB), hosted by the Biodiversity Institute of Ontario (BIO) at the University of Guelph, Ontario, Canada, processed the tissues samples; all operations were carried out following the standard high-throughput protocols in place at CCDB and available from http://ccdb.ca/resources/. For PCR amplification, we used a primer cocktail combining the LCO1490/HCO2198 pair (Folmer et al. 1994) with the LepF1/LepR1 pair (Hebert et al. 2003) for amplification of the full-length (658 bp) DNA barcode region of the COI gene.

## Geographic coverage

### Description

**Figure 1.**
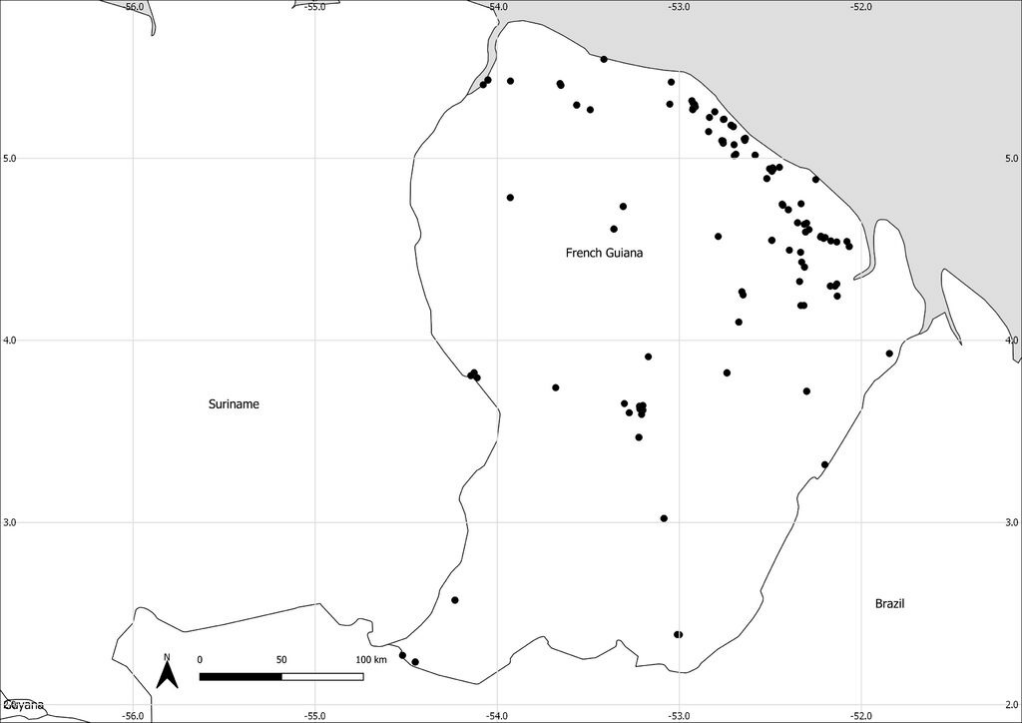
Distribution of the MANGF library records (Suppl. material 1 & Suppl. material 2).

The MANGF library covers all the administrative regions of French Guiana. The map in Fig. 1 represents the distribution of the MANGF records.

### Coordinates

2.1 and 5.8 Latitude; -51.6 and -54.6 Longitude.

## Taxonomic coverage

### Description

The MANGF library comprises 425 records for Mantodea from French Guiana belonging to eight different families. They represent 68 species in 42 genera. Table 1 provides the details for each family.

The nomenclature used generally follows that in TAXREF (TAXREF 2025), a taxonomic database that provides species names for biodiversity in French territories. New names and nomenclatural changes published after publication of the book of Moulin (2025) were adopted in the MANGF library. This strategy favours the consistency of names used within several independently constructed libraries in BOLD rather than an authoritative stand for one or another of alternative names. This should prevent, or at least limit, the existence of “parallel taxonomies” (multiple names or combination of names for a single species) in BOLD.

## Usage licence

### Usage licence

Open Data Commons Attribution License

## Data resources

### Data package title

MANGF DNA barcode reference library

### Resource link

https://doi.org/10.5883/DS-MANGF

### Alternative identifiers

MANGF library

### Number of data sets

1

### Data set 1.

#### Data set name

DS-MANGF

#### Data format

xml, tsv, fasta, ab1

#### Download URL

https://portal.boldsystems.org/recordset/DS-MANGF

#### Description

The MANGF library dataset can be downloaded from the Public Data Portal of BOLD in different formats (data as xml or tsv files, sequences and trace files as fasta and ab1 files). Alternatively, BOLD users can login and access the dataset via the Workbench platform of BOLD (see the public dataset list in the User Console page, under the name of first author); all records are also searchable within BOLD using the search function of the database.

The version of the library at the time of writing of this manuscript is also included as Suppl. materials 1, 2, 3 with record information in a CSV format and all aligned sequences in a fasta file.

**Data set 1.** 

Column label	Column description
processid	Unique identifier for the DNA sample.
sampleid	Unique identifier for the specimen and, by extension, the tissue sample used for DNA analysis.
fieldid	Identifier for specimen assigned in the field. Unless comments are added, it’s the same identifier as sampleid.
Bin	Barcode Index Number.
Museumid	Identifier for specimen of the museum of origin.
institution_storing	The full name of the institution that has physical possession of the voucher specimen.
Phylum	Phylum name.
Class	Class name.
Order	Order name.
Family	Family name.
Subfamily	Subfamily name.
Genus	Genus name.
Species	Species name.
Identifier	Full name of the person who identified the specimen.
collectors	The full or abbreviated names of the people or team responsible for collecting the sample in the field.
collectiondate	The date at which the sample was collected.
lifestage	The age class or life stage of the specimen at the time of sampling.
sex	The sex of the specimen.
reproduction	The presumed method of reproduction.
extrainfo	A brief note or project term associated with the specimen for rapid analysis.
notes	General notes regarding the specimen.
Lat	The geographic latitude (in decimal degrees) of the geographic centre of the sampling location.
Lon	The geographic longitude (in decimal degrees) of the geographic centre of the sampling location.
Coordinate_accuracy	A decimal representation of the accuracy of the coordinates given in the decimal Latitude and decimal Longitude.
Elev	Elevation of sampling site. Measured in metres relative to sea level. Negative values indicate a position below sea level.
Country	The full, unabbreviated name of the country, major political unit or ocean in which the organism was collected.
Sector	The full, unabbreviated name of the lake, conservation area or sector of park in which the organism was collected.
Exact site	Additional text descriptions regarding the exact location of the collection site relative to a geographic or biologically relevant landmark.

## Additional information

In the following sections, we provide a quick description of the results of DNA barcode analyses as carried out using the analytical tools available through BOLD’s workbench at the time of preparing this manuscript.

### Sequence composition

The summary statistics for nucleotide frequency distribution are provided in Table 2. The range of variation in GC content (27 – 37%) within our less diverse set of taxa (8 families) is large and similar to previous reports in insects (Clare et al. 2008). It is most variable at the 3^rd^ (2.7 – 22.7%) codon positions.

### Analyses of genetic distances

All sequence analyses were carried out in BOLD using Kimura-2 parameters (K2P) distances with BOLD handling the sequence alignment.

**Figure 2.**
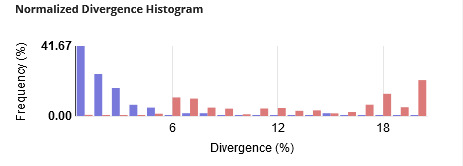
Frequency distribution of within-species (normalised, in violet, 60 species used) and within-genus (red, 40 species used) K2P distances for records of the MANGF library (sequences longer than 200 bp only: 424 records, 68 species). Table of distances is provided as Suppl. material 5 and Suppl. material 6.

**Figure 3.**
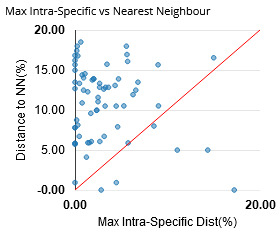
Scatterplot representing for each species of the MANGF library (sequences longer than 200 bp only: 68 species) the minimum distance to Nearest Neighbour (NN) plotted against the maximum intra-specific distance (Suppl. material 7).

All 425 sequences of the library were used to build a Neighbour-Joining (NJ) tree as illustrated in Suppl. material 4. For the analysis of intraspecific and interspecific distances, we reduced the dataset to sequences longer than 200 bp (424 records, 68 species). General summary statistics at the species, genus and family levels are given in Table 3; Fig. 2 shows the frequency distribution of genetic distances within species (normalised) and within genus. Fig. 3 represents the distribution of maximum intraspecific distances (singletons excluded) plotted against distances to Nearest Neighbour within the library. Overall, the Neighbour-Joining analysis resulted in a tree with most species forming distinct, cohesive units displaying minimal sequence variation (Suppl. material 4).

### Discrepancies between current taxonomy and DNA barcode results

While we are aware of the limits of our dataset to address taxonomic questions in cases where DNA barcodes and current taxonomy reveal a possible discordance, we report here some obvious conflicts between the results from DNA barcode analyses and species identifications derived from morphology.

High intraspecific divergence (> 4%) was observed within many genera (Table 4). These cases probably require further sampling and investigation to determine if they represent cases of overlooked or cryptic diversity or if they may represent geographical population structure, ancestral polymorphisms or variation resulting from *Wolbachia* infections (Smith et al. 2012 , Raupach et al. 2022). Indeed, an adult male specimen of the genus *Microphotina* was collected from the rocky outcrop of Armontabo (St. Georges sector), in February 2017 and has a sequence that differs widely from other known species of the genus in French Guiana. Low interspecific divergence (< 2%) is also observed between a pair of species belonging to *Microphotina* genus (Table 5). Here again, this case confirms the need for more sampling and further investigation to understand if our results reflect case of overlooked synonymy, introgression through past or ongoing hybridisation or recent speciation resulting in low level of divergence.

High intraspecific divergence is also observed within the *Chaeteessa* genus, which requires a sampling effort to delimit the species more precisely; moreover, the genitalia of males differ significantly (Moulin 2025).

Finally, an adult female specimen of the *Liturgusa* genus, collected in the Mitaraka mountains in 2015, shows a sequence very isolated from any other species of the genus known from French Guiana (unpublished sequences from MANGF BOLD project). Additionally, sampling efforts should be made to improve the delimitation of species.

## Supplementary Material

D8454430-C2D5-56FE-A1D9-44E27502FA7D 10.3897/BDJ.13.e149486.suppl1 Supplementary material 1 MANGF library - sequence data Data type Record information - sequence summary Brief description This CSV includes information about all records in BOLD for the MANGF library at the time of writing. It contains sequence information. File: oo_1297597.csv https://binary.pensoft.net/file/1297597 Moulin N.

DBBC859D-A380-5C12-A5C7-10944D6A3E18 10.3897/BDJ.13.e149486.suppl2 Supplementary material 2 MANGF library - specimen data Data type Record information - specimen data Brief description This CSV includes information about all records in BOLD for the MANGF library at the time of writing. It contains specimen data. File: oo_1297598.csv https://binary.pensoft.net/file/1297598 Moulin N.

271D4244-644F-5F0A-BBBC-E47519ECC778 10.3897/BDJ.13.e149486.suppl3 Supplementary material 3 MANGF library - DNA sequences Data type Genomic data, DNA sequences Brief description Sequences in fasta format for the fragment of the COI mtDNA gene used as a standard DNA barcode in animals. Each sequence is identified by a chain of characters consisting of, in the following order and separated by pipes: ProcessID, taxon_name, sampleID, Family_name, Genus_name, species_name, sex, stage_lifecycle, sector_name, BIN File: oo_1297600.fas https://binary.pensoft.net/file/1297600 Moulin N.

C1F68C18-3D49-59B4-8177-E39CB4121400 10.3897/BDJ.13.e149486.suppl4 Supplementary material 4 Neighbour-Joining tree reconstructed from the 424 DNA barcodes of the MANGF library Data type Distance tree Brief description NJ tree resulting from the analysis with BOLD of the 424 DNA barcode sequences of the MANGF library. Parameters for tree reconstruction are as follows: distance model: Kimura 2 Parameter; alignement method: BOLD aligner; sequence length: > 200 bp; pairwise deletion option; all three codon positions included. File: oo_1297601.pdf https://binary.pensoft.net/file/1297601 Moulin N.

3FBB2D8A-BDDC-5ADE-B800-4FE9834C3F12 10.3897/BDJ.13.e149486.suppl5 Supplementary material 5 Pairwise K2P distances within species Data type Genetic distances. Brief description This table lists K2P distances for all pairwise comparisons between conspecific records in the MANGF library (only DNA barcodes longer than 200 bp); distances are calculated in BOLD (https://www.boldsystems.org/). File: oo_1297602.csv https://binary.pensoft.net/file/1297602 Moulin N.

25C10293-FB84-5419-9EAA-717BA19838DC 10.3897/BDJ.13.e149486.suppl6 Supplementary material 6 Pairwise K2P distances within genera Data type Genetic distances. Brief description For the MANGF library (only DNA barcodes longer than 200 bp). This table lists K2P distances for all pairwise comparisons between heterospecific records of the same genus; distances are calculated in BOLD (https://www.boldsystems.org/). File: oo_1297603.csv https://binary.pensoft.net/file/1297603 Moulin N.

55E5FFDF-F211-58D3-9F89-99650C96DBE0 10.3897/BDJ.13.e149486.suppl7 Supplementary material 7 Intra-specific distances and distances to Nearest Neighbour (NN) Data type Genetic distances. Brief description This table provides, for each species of the MANGF library with sequences longer than 200 bp, mean and maximum intraspecific distances (non-applicable (N/A) for species represented as singletons in our dataset) as well as the distance to Nearest Neighbour (NN) within the library and its identification. File: oo_1297604.csv https://binary.pensoft.net/file/1297604 Moulin N.

## Figures and Tables

**Table 1.** Taxonomic coverage of the MANGF library giving details of the number of records, genera and species sampled within each of the eight families included (ordered alphabetically).

**Family**	**Records**	**Genera**	**Species**
Acanthopidae	76	14	16
Angelidae	50	1	7
Chaeteessidae	12	1	3
Liturgusidae	19	2	5
Mantidae	133	11	19
Mantoididae	22	2	2
Photinaidae	68	4	9
Thespidae	45	7	7
Total	425	42	68

**Table 2.** Nucleotide frequency distribution for sequences (> 200 bp, 424 sequences analysed) in the MANGF library.

	Min	Mean	Max	SE
G %	10.81	14.94	17.01	0.03
C %	13.13	17.54	22.96	0.07
A %	28.12	30.87	35.52	0.06
T %	30.00	36.66	39.54	0.07
GC %	27.76	32.47	37.23	0.08
GC % Codon Pos 1	40.00	44.64	51.11	0.08
GC % Codon Pos 2	37.25	41.60	45.62	0.05
GC % Codon Pos 3	2.73	11.27	22.73	0.21

**Table 3.** Summary of distance (K2P) variations at species, genus and family levels, as calculated with BOLD from 424 records of the MANGF library with DNA barcodes longer than 200 bp.

	n	Taxa	Comparisons	Min Dist (%)	Mean Dist (%)	Max Dist (%)	SE Dist (%)
Within Species	416	60	1851	0.00	1.80	50.00	0.00
Within Genus	262	14	2144	0.00	13.40	21.69	0.00
Within Family	362	6	12550	5.71	15.13	50.00	0.00

**Table 4.** List of species within the MANGF library (sequence length> 200 bp; 424 records, 68 species) with more than 4% intraspecific divergence (n = number of records).

Family	Species	n	Max. Intrasp. (%)
Acanthopidae	*Metiliabrunnerii*	8	6.80
Angelidae	*Angelaquinquemaculata*	2	6.57
Chaeteessidae	*Chaeteessacaudata*	5	6.27
Chaeteessidae	*Chaeteessafilata*	2	14.35
Chaeteessidae	*Chaeteessavalida*	5	11.06
Liturgusidae	*Liturgusamilleri*	5	4.45
Mantidae	*Alangularismultilobata*	8	5.74
Mantidae	*Choeradodisstrumaria*	4	4.34
Mantidae	*Heterovatespardalina*	7	4.61
Mantidae	*Stagmatopterasupplicaria*	9	8.53
Mantoididae	*Mantoidabrunneriana*	15	8.98
Mantoididae	*Vespamantoidatoulgoeti*	7	5.62
Photinaidae	*Microphotinaviridescens*	19	17.21
Photinaidae	*Photinapilosa*	10	9.02
Photinaidae	*Photinaovata*	11	5.28
Thespidae	*Bantiafusca*	11	4.71
Thespidae	*Dougonyxmaculosus*	9	5.56
Thespidae	*Macromusoniamajor*	7	5.69
Thespidae	*Pseudomiopteryxdispar*	9	14.99

**Table 5.** List of species pairs within the MANGF library for which the minimum distance to the nearest heterospecific record is below 2% (number of records for each taxon is given within brackets next to its name).

Family	Species pairs	Min. intersp. (%)
Photinaidae	*Microphotinavitripennis* (9) / *M.viridescens* (19)	0

## References

[B12689522] Bilousov O., Chaplinska M., Zhuk O., Gorobchyshyn V., Kozeretska I. (2011). *Mantisreligiosa* (Dyctioptera, Mantidae) Infected by Wolbachia. Vestnik Zoologii.

[B12485727] Boucher Stéphanie, Savage Jade (2022). ﻿DNA barcoding of the leaf-miner flies (Diptera, Agromyzidae) of Mitaraka, French Guiana. ZooKeys.

[B12485736] Brandon-Mong G. J., Gan H. M., Sing K. W., Lee P. S., Lim P. E., Wilson J. J. (2015). DNA metabarcoding of insects and allies: an evaluation of primers and pipelines. Bulletin of Entomological Research.

[B12485747] Brannoch Sydney K., Wieland Frank, Rivera Julio, Klass Klaus-Dieter, Béthoux Olivier, Svenson Gavin J. (2017). Manual of praying mantis morphology, nomenclature, and practices (Insecta, Mantodea). ZooKeys.

[B12485767] Carolan J. C., Murray T. E., Fitzpatrick Úna, Crossley John, Schmidt Hans, Cederberg Björn, McNally Luke, Paxton R. J., Williams P. H., Brown M. J. F. (2012). Colour patterns do not diagnose species: quantitative evaluation of a DNA barcoded cryptic Bumblebee complex. PLOS One.

[B12485782] Cheng Zhentao, Li Qiang, Deng Jun, Liu Qian, Huang Xiaolei (2023). The devil is in the details: Problems in DNA barcoding practices indicated by systematic evaluation of insect barcodes. Frontiers in Ecology and Evolution.

[B12689560] Christin Pascal-Antoine, Weinreich Daniel M., Besnard Guillaume (2010). Causes and evolutionary significance of genetic convergence. Trends in Genetics.

[B12485792] Clare E. L., Kerr K. C. R., von Königslöw T. E., Wilson J. J., Hebert P. D. N. (2008). Diagnosing Mitochondrial DNA Diversity: Applications of a Sentinel Gene Approach. Journal of Molecular Evolution.

[B12485802] Decaëns Thibaud, Bénéluz Frédéric, Ballesteros-Mejia Liliana, Bonilla Diego, Rougerie Rodolphe (2021). Description of three new species of *Automeris* Hübner, 1819 from Colombia and Brazil (Lepidoptera, Saturniidae, Hemileucinae). ZooKeys.

[B12485830] Dupont L., Porco D., Symondson W. O. C., Roy V. (2016). Hybridization relics complicate barcode‐based identification of species in earthworms. Molecular Ecology Resources.

[B12485839] Engel Michael S, Ceríaco Luis M P, Daniel Gimo M, Dellapé Pablo M, Löbl Ivan, Marinov Milen, Reis Roberto E, Young Mark T, Dubois Alain, Agarwal Ishan, Lehmann A. Pablo, Alvarado Mabel, Alvarez Nadir, Andreone Franco, Araujo-Vieira Katyuscia, Ascher John S, Baêta Délio, Baldo Diego, Bandeira Suzana A, Barden Phillip, Barrasso Diego A, Bendifallah Leila, Bockmann Flávio A, Böhme Wolfgang, Borkent Art, Brandão Carlos R F, Busack Stephen D, Bybee Seth M, Channing Alan, Chatzimanolis Stylianos, Christenhusz Maarten J M, Crisci Jorge V, D’elía Guillermo, Da Costa Luis M, Davis Steven R, De Lucena Carlos Alberto S, Deuve Thierry, Fernandes Elizalde Sara, Faivovich Julián, Farooq Harith, Ferguson Adam W, Gippoliti Spartaco, Gonçalves Francisco M P, Gonzalez Victor H, Greenbaum Eli, Hinojosa-Díaz Ismael A, Ineich Ivan, Jiang Jianping, Kahono Sih, Kury Adriano B, Lucinda Paulo H F, Lynch John D, Malécot Valéry, Marques Mariana P, Marris John W M, Mckellar Ryan C, Mendes Luis F, Nihei Silvio S, Nishikawa Kanto, Ohler Annemarie, Orrico Victor G D, Ota Hidetoshi, Paiva Jorge, Parrinha Diogo, Pauwels Olivier S G, Pereyra Martín O, Pestana Lueji B, Pinheiro Paulo D P, Prendini Lorenzo, Prokop Jakub, Rasmussen Claus, Rödel Mark-Oliver, Rodrigues Miguel Trefaut, Rodríguez Sara M, Salatnaya Hearty, Sampaio Íris, Sánchez-García Alba, Shebl Mohamed A, Santos Bruna S, Solórzano-Kraemer Mónica M, Sousa Ana C A, Stoev Pavel, Teta Pablo, Trape Jean-François, Dos Santos Carmen Van-Dúnem, Vasudevan Karthikeyan, Vink Cor J, Vogel Gernot, Wagner Philipp, Wappler Torsten, Ware Jessica L, Wedmann Sonja, Zacharie Chifundera Kusamba (2021). The taxonomic impediment: a shortage of taxonomists, not the lack of technical approaches. Zoological Journal of the Linnean Society.

[B12485937] Folmer O, Black M, Hoeh W, Lutz R, Vrijenhoek R (1994). DNA primers for amplification of mitochondrial cytochrome c oxidase subunit I from diverse metazoan invertebrates.. Molecular Marine Biology and Biotechnology.

[B12485956] François Alexandre, Roy Roger (2015). Le genre *Microphotina* Beier, 1935 : deux espèces, ou une seule ? (Mantodea, Photinaidae). Bulletin de la Société Entomologique de France.

[B12485965] Gaytán Álvaro, Bergsten Johannes, Canelo Tara, Pérez‐Izquierdo Carlos, Santoro Maria, Bonal Raul (2020). DNA Barcoding and geographical scale effect: The problems of undersampling genetic diversity hotspots. Ecology and Evolution.

[B12485976] Gemmell A. P., Borchers T. E., Marcus J. M. (2014). Molecular Population Structure of *Junonia* Butterflies from French Guiana, Guadeloupe, and Martinique. Psyche: A Journal of Entomology.

[B12486041] Govorov Valeriy, Shcherbakov Evgeny, Janšta Petr, Černá B. Barbora (2024). First assessment of the biodiversity of praying mantises (Insecta: Mantodea) in Cameroon with DNA barcoding. PLOS One.

[B12486050] Guitet Stéphane, Pélissier Raphaël, Brunaux Olivier, Jaouen Gaëlle, Sabatier Daniel (2015). Geomorphological landscape features explain floristic patterns in French Guiana rainforest. Biodiversity and Conservation.

[B12486069] Hebert P. D.N., Ratnasingham Sujeevan, de Waard Jeremy R. (2003). Barcoding animal life: cytochrome *c* oxidase subunit 1 divergences among closely related species. Proceedings of the Royal Society of London. Series B: Biological Sciences.

[B12486060] Hebert P. D. N., Cywinska Alina, Ball Shelley L., deWaard Jeremy R. (2003). Biological identifications through DNA barcodes. Proceedings of the Royal Society of London. Series B: Biological Sciences.

[B12486078] Hebert P. D. N., Bock D. G., Prosser S. W. J. (2023). Interrogating 1000 insect genomes for NUMTs: A risk assessment for estimates of species richness. PLOS One.

[B12486088] Janzen Daniel H., Hallwachs Winnie, Blandin Patrick, Burns John M., Cadiou Jean‐Marie, Chacon Isidro, Dapkey Tanya, Deans Andrew R., Epstein Marc E., Espinoza Bernardo, Franclemont John G., Haber William A., Hajibabaei Mehrdad, Hall J. P. W., Hebert P. D. N., Gauld I. D., Harvey D. J., Hausmann A., Kitching I. J., Lafontaine D., Landry J. ‐F., Lemaire C., Miller J. Y., Miller J. S., Miller LEE, Miller S. E., Montero JOSE, Munroe EUGENE, Green SUZANNE RAB, Ratnasingham SUJEEVAN, Rawlins JOHN E., Robbins ROBERT K., Rodriguez JOSEPHINE J., Rougerie RODOLPHE, Sharkey MICHAEL J., Smith M. ALEX, Solis M. ALMA, Sullivan J. BOLLING, Thiaucourt PAUL, Wahl DAVID B., Weller SUSAN J., Whitfield JAMES B., Willmott KEITH R., Wood D. MONTY, Woodley NORMAN E., Wilson JOHN J. (2009). Integration of DNA barcoding into an ongoing inventory of complex tropical biodiversity. Molecular Ecology Resources.

[B12689540] Jiménez‐Florido Patricia, Aquilino Mónica, Buckley David, Bella José L., Planelló Rosario (2024). Differential gene expression in *Chorthippusparallelus* (Zetterstedt, 1821) (Orthoptera: Acrididae: Gomphocerinae) induced by *Wolbachia* infection. Insect Science.

[B12486140] Jordal B. H., Kambestad Marius (2013). DNA barcoding of bark and ambrosia beetles reveals excessive NUMTs and consistent east‐west divergence across Palearctic forests. Molecular Ecology Resources.

[B12486149] Kajtoch Łukasz, Kolasa Michał, Kubisz Daniel, Gutowski J. M., Ścibior Radosław, Mazur M. A., Holecová Milada (2019). Using host species traits to understand the Wolbachia infection distribution across terrestrial beetles. Scientific Reports.

[B12486162] Klopfstein Seraina, Kropf Christian, Baur Hannes (2016). *Wolbachiaendosymbionts* distort DNA barcoding in the parasitoid wasp genus *Diplazon* (Hymenoptera: Ichneumonidae). Zoological Journal of the Linnean Society.

[B12486172] Kolasa M., Kubisz D., Mazur MA., Ścibior R., Kajtoch Ł. (2018). *Wolbachia* prevalence and diversity in selected riverine predatory beetles (Bembidiini and Paederini).. Bulletin of Insectology.

[B12486184] Leite Luis Anderson Ribeiro (2012). Mitochondrial pseudogenes in insect DNA barcoding: differing points of view on the same issue. Biota Neotropica.

[B12486193] Liu Qinpeng, Liu Yingqi, Liu Qiaoqiao, Tian Li, Li Hu, Song Fan, Cai Wanzhi (2023). Exploring the mitogenomes of Mantodea: New insights from structural diversity and higher-level phylogenomic analyses. International Journal of Molecular Sciences.

[B12486216] Maggia Marie-Eugénie, Decaëns Thibaud, Lapied Emmanuel, Dupont Lise, Roy Virginie, Schimann Heidy, Orivel Jérôme, Murienne Jérôme, Baraloto Christopher, Cottenie Karl, Steinke Dirk (2021). At each site its diversity: DNA barcoding reveals remarkable earthworm diversity in neotropical rainforests of French Guiana. Applied Soil Ecology.

[B12486205] Ma Yue, Zhang L. P., Lin Y. J., Yu D. N., Storey K. B., Zhang J. Y. (2023). Phylogenetic relationships and divergence dating of Mantodea using mitochondrial phylogenomics. Systematic Entomology.

[B12486232] Mittermeier R. A., Mittermeier C. G., Brooks T. M., Pilgrim J. D., Konstant W. R., da Fonseca G. A. B., Kormos C. (2003). Wilderness and biodiversity conservation. Proceedings of the National Academy of Sciences.

[B12486244] Moulin Nicolas, Decaëns Thibaud, Annoyer Philippe (2017). Diversity of mantids (Dictyoptera: Mantodea) of Sangha-Mbaere Region, Central African Republic, with some ecological data and DNA barcoding. Journal of Orthoptera Research.

[B12486253] Moulin Nicolas (2020). A cryptic new species of *Chlidonoptera* Karsch, 1892 from the south west protected zone of the Central African Republic (Insecta, Mantodea, Hymenopodidae). ZooKeys.

[B12486363] Moulin Nicolas, Roy Roger (2020). Synthèse des connaissances des Mantodea de Guyane. Naturae.

[B12486262] Moulin Nicolas (2023). The genus *Vates* Burmeister, 1838, in French Guiana, with the description of two new species (Mantodea, Mantidae, Vatinae). Bulletin de la Société entomologique de France.

[B12486379] Moulin Nicolas, Schwarz Christian J. (2023). Two new genera of Acanthopidae (Mantodea) from the Amazon region, with description of a new species. Zoosystema.

[B12486271] Moulin N. (2025). Les Mantodea de Guyane. Insecta, Polyneoptera.

[B12486388] Moulton M. J., Song HOJUN, Whiting M. F. (2010). Assessing the effects of primer specificity on eliminating numt coamplification in DNA barcoding: a case study from Orthoptera (Arthropoda: Insecta). Molecular Ecology Resources.

[B12487191] Mutanen Marko, Kivelä S. M., Vos R. A., Doorenweerd Camiel, Ratnasingham Sujeevan, Hausmann Axel, Huemer Peter, Dincă Vlad, van Nieukerken E. J., Lopez-Vaamonde Carlos, Vila Roger, Aarvik Leif, Decaëns Thibaud, Efetov K. A., Hebert P. D. N., Johnsen Arild, Karsholt Ole, Pentinsaari Mikko, Rougerie Rodolphe, Segerer Andreas, Tarmann Gerhard, Zahiri Reza, Godfray H. C. J. (2016). Species-level para- and polyphyly in DNA barcode gene trees: Strong operational bias in European Lepidoptera. Systematic Biology.

[B12486397] Myers Norman, Mittermeier Russell A., Mittermeier Cristina G., da Fonseca Gustavo A. B., Kent Jennifer (2000). Biodiversity hotspots for conservation priorities. Nature.

[B12486407] Nabholz Benoit (2023). Incomplete lineage sorting explains the low performance of DNA barcoding in a radiation of four species of Western European grasshoppers (Orthoptera: Acrididae: Chorthippus). Biological Journal of the Linnean Society.

[B12486416] Otte D., Spearman L., Stiewe M. Mantodea Species File Online. http://Mantodea.SpeciesFile.org.

[B12486424] Ožana Stanislav, Dolný Aleš, Pánek Tomáš (2022). Nuclear copies of mitochondrial DNA as a potential problem for phylogenetic and population genetic studies of Odonata. Systematic Entomology.

[B12486433] Ratnasingham SUJEEVAN, Hebert P. D. N. (2007). bold: The Barcode of Life Data System (http://www.barcodinglife.org). Molecular Ecology Notes.

[B12689550] Raupach Michael J., Rulik Björn, Spelda Jörg (2022). Surprisingly high genetic divergence of the mitochondrial DNA barcode fragment (COI) within Central European woodlice species (Crustacea, Isopoda, Oniscidea). ZooKeys.

[B12486450] Rongier Gaëtan, Sagne Audrey, Etienne Sandrine, Petitclerc Frederic, Jaouen Gaelle, Murienne Jerome, Orivel Jerome (2023). Ants of French Guiana: 16S rRNA sequence dataset. Biodiversity Data Journal.

[B12486462] Rougerie R., Haxaire Jean, Kitching I. J., Hebert P. D. N. (2012). DNA barcodes and morphology reveal a hybrid hawkmoth in Tahiti (Lepidoptera: Sphingidae). Invertebrate Systematics.

[B12486471] Rougerie Rodolphe, Lopez-Vaamonde Carlos, Barnouin Thomas, Delnatte Julien, Moulin Nicolas, Noblecourt Thierry, Nusillard Benoît, Parmain Guillem, Soldati Fabien, Bouget Christophe (2015). PASSIFOR: A reference library of DNA barcodes for French saproxylic beetles (Insecta, Coleoptera). Biodiversity Data Journal.

[B12486486] Rougerie R., Sire L., Lévêque A., Nicolas Violaine (2024). L’inventaire de la biodiversité aujourd’hui – nouvelles méthodes et découvertes..

[B12486500] Roy Roger (2002). Une remarquable espèce nouvelle d'*Acanthops* Audinet-Serville, 1831, en Guyane française (Dictyoptera, Mantodea). Bulletin de la Société entomologique de France.

[B12486509] Roy Roger (2002). Révision du genre néotropical *Macromantis* Saussure, 1871 (Dictyoptera, Mantidae). Bulletin de la Société entomologique de France.

[B12486518] Roy R. (2002). Commentaires à propos du genre *Plesiacanthops* Chopard, 1913, et redescription d’*Acanthopstuberculata* Saussure, 1870 (Dictyoptera, Mantodea).. Revue Française d'Entomologie (N.S.)..

[B12486527] Roy Roger (2003). *Callivatesstephanei* n. gen. n. sp. des Guyanes (Dictyoptera, Mantidae). Bulletin de la Société Entomologique de France.

[B12486536] Roy Roger (2004). Révision et phylogénie des Choeradodini Kirby, 1904 (Dictyoptera, Mantidae). Bulletin de la Société entomologique de France.

[B12486545] Roy Roger (2004). *Lagrecacanthops* et *Miracanthops*, deux nouveaux genres d'Acanthopinae (Dictyoptera, Mantodea, Acanthopidae). Bulletin de la Société Entomologique de France.

[B12486554] Roy Roger (2005). Mises au point sur le genre *Stenophylla* Westwood (Dictyoptera, Acanthopidae). Bulletin de la Société entomologique de France.

[B12486563] Roy Roger (2006). Vue d'ensemble sur les Acontistinae Giglio-Tos, 1919 (Dictyoptera, Acanthopidae). Bulletin de la Société entomologique de France.

[B12486572] Roy R., Ehrmann R. (2009). Révision du genre *Zoolea* Audinet-Serville [Mantodea, Mantidae, Vatinae].. Revue française d’Entomologie (N.S.)..

[B12486581] Roy Roger (2010). Contribution à la connaissance du genre néotropical *Mantoida* Newman, 1838 (Dict., Mantoididae). Bulletin de la Société entomologique de France.

[B12486590] Roy Roger (2011). Redécouverte en Guyane d'*Angelamaxima* (Chopard, 1910) (Dict., Mantidae). Bulletin de la Société entomologique de France.

[B12486599] Roy Roger (2012). Une curieuse ressemblance entre des espèces des genres voisins *Zoolea* Audinet Serville, 1838, et *Vates* Burmeister, 1838 (Dict., Mantidae, Vatinae). Bulletin de la Société entomologique de France.

[B12486608] Roy Roger (2015). Un nouveau genre de Mante de Guyane française (Mantodea, Acanthopidae). Bulletin de la Société entomologique de France.

[B12689569] Roy Roger, Schütte Kai (2016). Mises au point sur le genre *Brancsikia* Saussure & Zehntner (Mantodea, Epaphroditidae). Bulletin de la Société entomologique de France.

[B12486617] Roy Roger (2019). Les mantes (Dictyoptera, Mantodea) du massif du Mitaraka (Guyane). Zoosystema.

[B12689587] Saussure H., Zehntner L., Grandidier (1895). Histoire physique, naturelle et politique de Madagascar..

[B12486636] Sire Lucas, Schmidt Yáñez Paul, Bézier Annie, Courtial Béatrice, Mbedi Susan, Sparmann Sarah, Larrieu Laurent, Rougerie Rodolphe, Bouget Christophe, Monaghan Michael T., Herniou Elisabeth A., Lopez-Vaamonde Carlos (2023). Persisting roadblocks in arthropod monitoring using non-destructive metabarcoding from collection media of passive traps. PeerJ.

[B12486653] Smith M. Alex, Bertrand Claudia, Crosby Kate, Eveleigh E. S., Fernandez-Triana Jose, Fisher B. L., Gibbs Jason, Hajibabaei Mehrdad, Hallwachs Winnie, Hind Katharine, Hrcek Jan, Huang D. W., Janda Milan, Janzen D. H., Li Yanwei, Miller S. E., Packer Laurence, Quicke Donald, Ratnasingham Sujeevan, Rodriguez Josephine, Rougerie Rodolphe, Shaw M. R., Sheffield Cory, Stahlhut J. K., Steinke Dirk, Whitfield James, Wood Monty, Zhou Xin (2012). Wolbachia and DNA barcoding insects: Patterns, potential, and problems. PLOS One.

[B12486686] Song Hojun, Buhay J. E., Whiting M. F., Crandall K. A. (2008). Many species in one: DNA barcoding overestimates the number of species when nuclear mitochondrial pseudogenes are coamplified. Proceedings of the National Academy of Sciences.

[B12486713] Talaga Stanislas, Leroy Céline, Guidez Amandine, Dusfour Isabelle, Girod Romain, Dejean Alain, Murienne Jérôme (2017). DNA reference libraries of French Guianese mosquitoes for barcoding and metabarcoding. PLOS One.

[B12486725] TAXREF TAXREF v18.0, référentiel taxonomique pour la France. PatriNat (OFB-CNRS-MNHN-IRD), Muséum national d’Histoire naturelle, Paris.. https://inpn.mnhn.fr/telechargement/referentielEspece/taxref/18.0/menu.

[B12485758] Touroult Julien, Stéphane Brûlé (2014). Insects of French Guiana: a baseline for diversity and taxonomic effort. ZooKeys.

[B12485821] Young Kenneth R. (2009). A Conservation Assessment of the Terrestrial Ecoregions of Latin America and the Caribbean By Eric Dinerstein, David M. Olson, Douglas J. Graham, Avis L. Webster, Steven A. Primm, Marnie P. Bookbinder and George Ledec xvii + 129 pp., 3 figs, 31 tables & 11 maps, 28 × 21.5 × 1 cm, ISBN 0 8213 3295 3 paperback, US$29.95, Washington, DC, USA: World Wildlife Fund and World Bank, 1995. Environmental Conservation.

[B12689578] Zhang JIA-ZHI, Price BENJAMIN (2024). Revision of the genus *Deroplatys* Westwood, 1839 (Mantodea: Deroplatyidae) with the description of three new species. Zootaxa.

