# Supplementary material for: MANGF: a reference library of DNA barcodes for Mantodea from French Guiana (Insecta, Dictyoptera)
Source: Biodivers Data J. 2025 Apr 9;13:e149486. doi: 10.3897/BDJ.13.e149486 (PMC12004074; doi:10.3897/BDJ.13.e149486)
Supplement: Supplementary material 4 — Neighbour-Joining tree reconstructed from the 424 DNA barcodes of the MANGF library [file bdj-13-e149486-s004.pdf]

BOLD TaxonID Tree

Title	:	Tree Result - DS-MANGF (425 records selected)
Date	:	25-Mar-2025
Data Type	:	Nucleotide
Distance Model	:	Kimura 2 Parameter
Marker	:	COI-5P
Colourization	:	[blue]=Stop Codons [red]=Contamination or misidentification

Label	:	Sample ID
Label	:	Family
Label	:	Species
Label	:	Sector
Label	:	Sequence Length

Sequence Count	:	415
Species count	:	66
Genus count	:	41
Family count	:	8
Unidentified	:	0

BIN Count	:	112

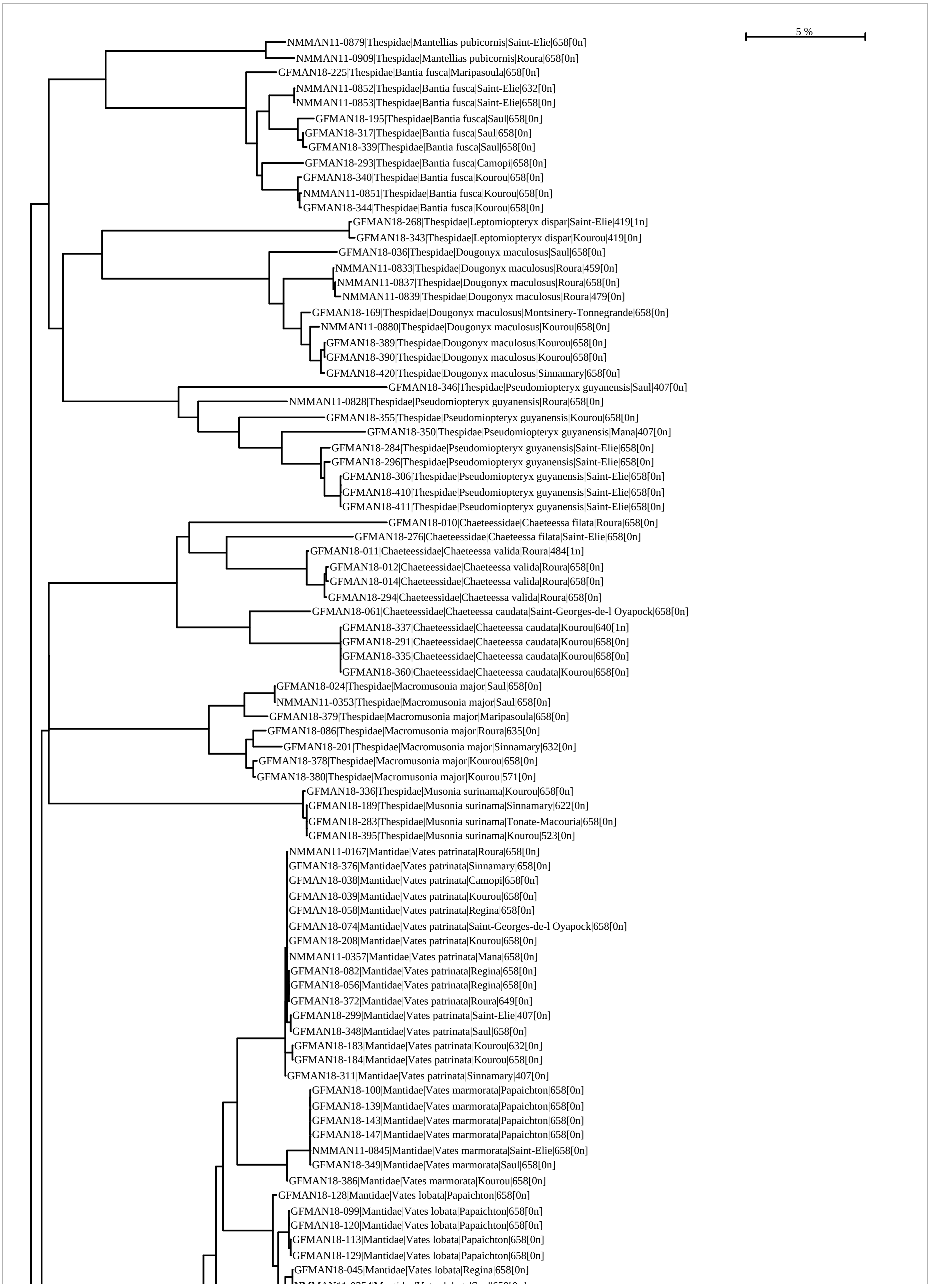
5 %
NMMAN11-0879|Thespidae|Mantellias pubicornis|Saint-Elie|658[0n]
NMMAN11-0909|Thespidae|Mantellias pubicornis|Roura|658[0n]
GFMAN18-225|Thespidae|Bantia fusca|Maripasoula|658[0n]
NMMAN11-0852|Thespidae|Bantia fusca|Saint-Elie|632[0n]
NMMAN11-0853|Thespidae|Bantia fusca|Saint-Elie|658[0n]
GFMAN18-195|Thespidae|Bantia fusca|Saul|658[0n]
GFMAN18-317|Thespidae|Bantia fusca|Saul|658[0n]
GFMAN18-339|Thespidae|Bantia fusca|Saul|658[0n]
GFMAN18-293|Thespidae|Bantia fusca|Camopi|658[0n]
GFMAN18-340|Thespidae|Bantia fusca|Kourou|658[0n]
NMMAN11-0851|Thespidae|Bantia fusca|Kourou|658[0n]
GFMAN18-344|Thespidae|Bantia fusca|Kourou|658[0n]
GFMAN18-268|Thespidae|Leptomiopteryx dispar|Saint-Elie|419[1n]
GFMAN18-343|Thespidae|Leptomiopteryx dispar|Kourou|419[0n]
GFMAN18-036|Thespidae|Dougonyx maculosus|Saul|658[0n]
NMMAN11-0833|Thespidae|Dougonyx maculosus|Roura|459[0n]
NMMAN11-0837|Thespidae|Dougonyx maculosus|Roura|658[0n]
NMMAN11-0839|Thespidae|Dougonyx maculosus|Roura|479[0n]
GFMAN18-169|Thespidae|Dougonyx maculosus|Montsinery-Tonnegrande|658[0n]
NMMAN11-0880|Thespidae|Dougonyx maculosus|Kourou|658[0n]
GFMAN18-389|Thespidae|Dougonyx maculosus|Kourou|658[0n]
GFMAN18-390|Thespidae|Dougonyx maculosus|Kourou|658[0n]
GFMAN18-420|Thespidae|Dougonyx maculosus|Sinnamary|658[0n]
GFMAN18-346|Thespidae|Pseudomiopteryx guyanensis|Saul|407[0n]
NMMAN11-0828|Thespidae|Pseudomiopteryx guyanensis|Roura|658[0n]
GFMAN18-355|Thespidae|Pseudomiopteryx guyanensis|Kourou|658[0n]
GFMAN18-350|Thespidae|Pseudomiopteryx guyanensis|Mana|407[0n]
GFMAN18-284|Thespidae|Pseudomiopteryx guyanensis|Saint-Elie|658[0n]
GFMAN18-296|Thespidae|Pseudomiopteryx guyanensis|Saint-Elie|658[0n]
GFMAN18-306|Thespidae|Pseudomiopteryx guyanensis|Saint-Elie|658[0n]
GFMAN18-410|Thespidae|Pseudomiopteryx guyanensis|Saint-Elie|658[0n]
GFMAN18-411|Thespidae|Pseudomiopteryx guyanensis|Saint-Elie|658[0n]
GFMAN18-010|Chaeteessidae|Chaeteessa filata|Roura|658[0n]
GFMAN18-276|Chaeteessidae|Chaeteessa filata|Saint-Elie|658[0n]
GFMAN18-011|Chaeteessidae|Chaeteessa valida|Roura|484[1n]
GFMAN18-012|Chaeteessidae|Chaeteessa valida|Roura|658[0n]
GFMAN18-014|Chaeteessidae|Chaeteessa valida|Roura|658[0n]
GFMAN18-294|Chaeteessidae|Chaeteessa valida|Roura|658[0n]
GFMAN18-061|Chaeteessidae|Chaeteessa caudata|Saint-Georges-de-l Oyapock|658[0n]
GFMAN18-337|Chaeteessidae|Chaeteessa caudata|Kourou|640[1n]
GFMAN18-291|Chaeteessidae|Chaeteessa caudata|Kourou|658[0n]
GFMAN18-335|Chaeteessidae|Chaeteessa caudata|Kourou|658[0n]
GFMAN18-360|Chaeteessidae|Chaeteessa caudata|Kourou|658[0n]
GFMAN18-024|Thespidae|Macromusonia major|Saul|658[0n]
NMMAN11-0353|Thespidae|Macromusonia major|Saul|658[0n]
GFMAN18-379|Thespidae|Macromusonia major|Maripasoula|658[0n]
GFMAN18-086|Thespidae|Macromusonia major|Roura|635[0n]
GFMAN18-201|Thespidae|Macromusonia major|Sinnamary|632[0n]
GFMAN18-378|Thespidae|Macromusonia major|Kourou|658[0n]
GFMAN18-380|Thespidae|Macromusonia major|Kourou|571[0n]
GFMAN18-336|Thespidae|Musonia surinama|Kourou|658[0n]
GFMAN18-189|Thespidae|Musonia surinama|Sinnamary|622[0n]
GFMAN18-283|Thespidae|Musonia surinama|Tonate-Macouria|658[0n]
GFMAN18-395|Thespidae|Musonia surinama|Kourou|523[0n]
NMMAN11-0167|Mantidae|Vates patrinata|Roura|658[0n]
GFMAN18-376|Mantidae|Vates patrinata|Sinnamary|658[0n]
GFMAN18-038|Mantidae|Vates patrinata|Camopi|658[0n]
GFMAN18-039|Mantidae|Vates patrinata|Kourou|658[0n]
GFMAN18-058|Mantidae|Vates patrinata|Regina|658[0n]
GFMAN18-074|Mantidae|Vates patrinata|Saint-Georges-de-l Oyapock|658[0n]
GFMAN18-208|Mantidae|Vates patrinata|Kourou|658[0n]
NMMAN11-0357|Mantidae|Vates patrinata|Mana|658[0n]
GFMAN18-082|Mantidae|Vates patrinata|Regina|658[0n]
GFMAN18-056|Mantidae|Vates patrinata|Regina|658[0n]
GFMAN18-372|Mantidae|Vates patrinata|Roura|649[0n]
GFMAN18-299|Mantidae|Vates patrinata|Saint-Elie|407[0n]
GFMAN18-348|Mantidae|Vates patrinata|Saul|658[0n]
GFMAN18-183|Mantidae|Vates patrinata|Kourou|632[0n]
GFMAN18-184|Mantidae|Vates patrinata|Kourou|658[0n]
GFMAN18-311|Mantidae|Vates patrinata|Sinnamary|407[0n]
GFMAN18-100|Mantidae|Vates marmorata|Papaichton|658[0n]
GFMAN18-139|Mantidae|Vates marmorata|Papaichton|658[0n]
GFMAN18-143|Mantidae|Vates marmorata|Papaichton|658[0n]
GFMAN18-147|Mantidae|Vates marmorata|Papaichton|658[0n]
NMMAN11-0845|Mantidae|Vates marmorata|Saint-Elie|658[0n]
GFMAN18-349|Mantidae|Vates marmorata|Saul|658[0n]
GFMAN18-386|Mantidae|Vates marmorata|Kourou|658[0n]
GFMAN18-128|Mantidae|Vates lobata|Papaichton|658[0n]
GFMAN18-099|Mantidae|Vates lobata|Papaichton|658[0n]
GFMAN18-120|Mantidae|Vates lobata|Papaichton|658[0n]
GFMAN18-113|Mantidae|Vates lobata|Papaichton|658[0n]
GFMAN18-129|Mantidae|Vates lobata|Papaichton|658[0n]
GFMAN18-045|Mantidae|Vates lobata|Regina|658[0n]

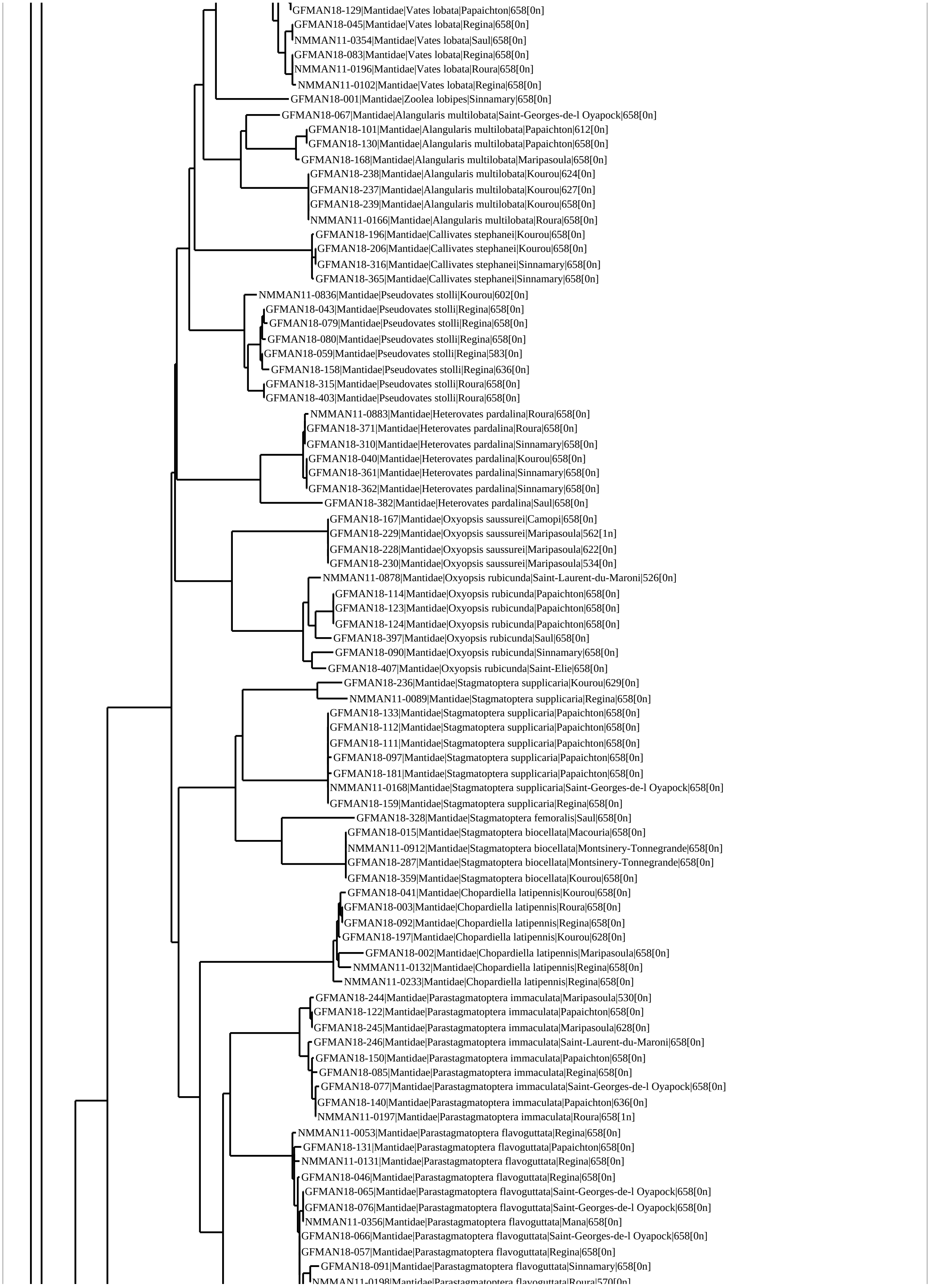
GFMAN18-129|Mantidae|Vates lobata|Papaichton|658[0n]
GFMAN18-045|Mantidae|Vates lobata|Regina|658[0n]
NMMAN11-0354|Mantidae|Vates lobata|Saul|658[0n]
GFMAN18-083|Mantidae|Vates lobata|Regina|658[0n]
NMMAN11-0196|Mantidae|Vates lobata|Roura|658[0n]
NMMAN11-0102|Mantidae|Vates lobata|Regina|658[0n]
GFMAN18-001|Mantidae|Zoolea lobipes|Sinnamary|658[0n]
GFMAN18-067|Mantidae|Alangularis multilobata|Saint-Georges-de-l Oyapock|658[0n]
GFMAN18-101|Mantidae|Alangularis multilobata|Papaichton|612[0n]
GFMAN18-130|Mantidae|Alangularis multilobata|Papaichton|658[0n]
GFMAN18-168|Mantidae|Alangularis multilobata|Maripasoula|658[0n]
GFMAN18-238|Mantidae|Alangularis multilobata|Kourou|624[0n]
GFMAN18-237|Mantidae|Alangularis multilobata|Kourou|627[0n]
GFMAN18-239|Mantidae|Alangularis multilobata|Kourou|658[0n]
NMMAN11-0166|Mantidae|Alangularis multilobata|Roura|658[0n]
GFMAN18-196|Mantidae|Callivates stephanei|Kourou|658[0n]
GFMAN18-206|Mantidae|Callivates stephanei|Kourou|658[0n]
GFMAN18-316|Mantidae|Callivates stephanei|Sinnamary|658[0n]
GFMAN18-365|Mantidae|Callivates stephanei|Sinnamary|658[0n]
NMMAN11-0836|Mantidae|Pseudovates stolli|Kourou|602[0n]
GFMAN18-043|Mantidae|Pseudovates stolli|Regina|658[0n]
GFMAN18-079|Mantidae|Pseudovates stolli|Regina|658[0n]
GFMAN18-080|Mantidae|Pseudovates stolli|Regina|658[0n]
GFMAN18-059|Mantidae|Pseudovates stolli|Regina|583[0n]
GFMAN18-158|Mantidae|Pseudovates stolli|Regina|636[0n]
GFMAN18-315|Mantidae|Pseudovates stolli|Roura|658[0n]
GFMAN18-403|Mantidae|Pseudovates stolli|Roura|658[0n]
NMMAN11-0883|Mantidae|Heterovates pardalina|Roura|658[0n]
GFMAN18-371|Mantidae|Heterovates pardalina|Roura|658[0n]
GFMAN18-310|Mantidae|Heterovates pardalina|Sinnamary|658[0n]
GFMAN18-040|Mantidae|Heterovates pardalina|Kourou|658[0n]
GFMAN18-361|Mantidae|Heterovates pardalina|Sinnamary|658[0n]
GFMAN18-362|Mantidae|Heterovates pardalina|Sinnamary|658[0n]
GFMAN18-382|Mantidae|Heterovates pardalina|Saul|658[0n]
GFMAN18-167|Mantidae|Oxyopsis saussurei|Camopi|658[0n]
GFMAN18-229|Mantidae|Oxyopsis saussurei|Maripasoula|562[1n]
GFMAN18-228|Mantidae|Oxyopsis saussurei|Maripasoula|622[0n]
GFMAN18-230|Mantidae|Oxyopsis saussurei|Maripasoula|534[0n]
NMMAN11-0878|Mantidae|Oxyopsis rubicunda|Saint-Laurent-du-Maroni|526[0n]
GFMAN18-114|Mantidae|Oxyopsis rubicunda|Papaichton|658[0n]
GFMAN18-123|Mantidae|Oxyopsis rubicunda|Papaichton|658[0n]
GFMAN18-124|Mantidae|Oxyopsis rubicunda|Papaichton|658[0n]
GFMAN18-397|Mantidae|Oxyopsis rubicunda|Saul|658[0n]
GFMAN18-090|Mantidae|Oxyopsis rubicunda|Sinnamary|658[0n]
GFMAN18-407|Mantidae|Oxyopsis rubicunda|Saint-Elie|658[0n]
GFMAN18-236|Mantidae|Stagmatoptera supplicaria|Kourou|629[0n]
NMMAN11-0089|Mantidae|Stagmatoptera supplicaria|Regina|658[0n]
GFMAN18-133|Mantidae|Stagmatoptera supplicaria|Papaichton|658[0n]
GFMAN18-112|Mantidae|Stagmatoptera supplicaria|Papaichton|658[0n]
GFMAN18-111|Mantidae|Stagmatoptera supplicaria|Papaichton|658[0n]
GFMAN18-097|Mantidae|Stagmatoptera supplicaria|Papaichton|658[0n]
GFMAN18-181|Mantidae|Stagmatoptera supplicaria|Papaichton|658[0n]
NMMAN11-0168|Mantidae|Stagmatoptera supplicaria|Saint-Georges-de-l Oyapock|658[0n]
GFMAN18-159|Mantidae|Stagmatoptera supplicaria|Regina|658[0n]
GFMAN18-328|Mantidae|Stagmatoptera femoralis|Saul|658[0n]
GFMAN18-015|Mantidae|Stagmatoptera biocellata|Macouria|658[0n]
NMMAN11-0912|Mantidae|Stagmatoptera biocellata|Montsinery-Tonnegrande|658[0n]
GFMAN18-287|Mantidae|Stagmatoptera biocellata|Montsinery-Tonnegrande|658[0n]
GFMAN18-359|Mantidae|Stagmatoptera biocellata|Kourou|658[0n]
GFMAN18-041|Mantidae|Chopardiella latipennis|Kourou|658[0n]
GFMAN18-003|Mantidae|Chopardiella latipennis|Roura|658[0n]
GFMAN18-092|Mantidae|Chopardiella latipennis|Regina|658[0n]
GFMAN18-197|Mantidae|Chopardiella latipennis|Kourou|628[0n]
GFMAN18-002|Mantidae|Chopardiella latipennis|Maripasoula|658[0n]
NMMAN11-0132|Mantidae|Chopardiella latipennis|Regina|658[0n]
NMMAN11-0233|Mantidae|Chopardiella latipennis|Regina|658[0n]
GFMAN18-244|Mantidae|Parastagmatoptera immaculata|Maripasoula|530[0n]
GFMAN18-122|Mantidae|Parastagmatoptera immaculata|Papaichton|658[0n]
GFMAN18-245|Mantidae|Parastagmatoptera immaculata|Maripasoula|628[0n]
GFMAN18-246|Mantidae|Parastagmatoptera immaculata|Saint-Laurent-du-Maroni|658[0n]
GFMAN18-150|Mantidae|Parastagmatoptera immaculata|Papaichton|658[0n]
GFMAN18-085|Mantidae|Parastagmatoptera immaculata|Regina|658[0n]
GFMAN18-077|Mantidae|Parastagmatoptera immaculata|Saint-Georges-de-l Oyapock|658[0n]
GFMAN18-140|Mantidae|Parastagmatoptera immaculata|Papaichton|636[0n]
NMMAN11-0197|Mantidae|Parastagmatoptera immaculata|Roura|658[1n]
NMMAN11-0053|Mantidae|Parastagmatoptera flavoguttata|Regina|658[0n]
GFMAN18-131|Mantidae|Parastagmatoptera flavoguttata|Papaichton|658[0n]
NMMAN11-0131|Mantidae|Parastagmatoptera flavoguttata|Regina|658[0n]
GFMAN18-046|Mantidae|Parastagmatoptera flavoguttata|Regina|658[0n]
GFMAN18-065|Mantidae|Parastagmatoptera flavoguttata|Saint-Georges-de-l Oyapock|658[0n]
GFMAN18-076|Mantidae|Parastagmatoptera flavoguttata|Saint-Georges-de-l Oyapock|658[0n]
NMMAN11-0356|Mantidae|Parastagmatoptera flavoguttata|Mana|658[0n]
GFMAN18-066|Mantidae|Parastagmatoptera flavoguttata|Saint-Georges-de-l Oyapock|658[0n]
GFMAN18-057|Mantidae|Parastagmatoptera flavoguttata|Regina|658[0n]
GFMAN18-091|Mantidae|Parastagmatoptera flavoguttata|Sinnamary|658[0n]
NMMAN11-0198|Mantidae|Parastagmatoptera flavoguttata|Roura|570[0n]

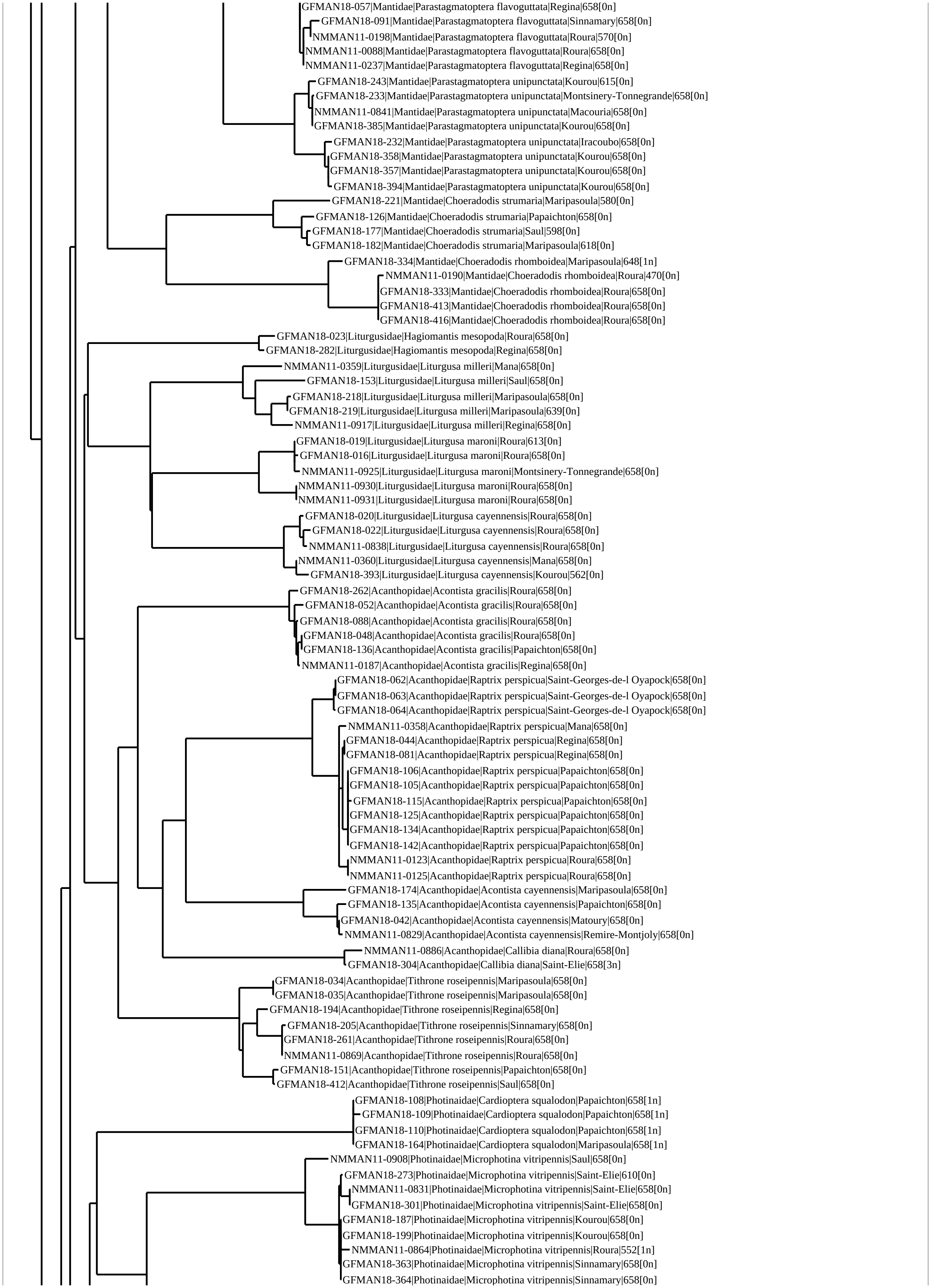
GFMAN18-057|Mantidae|Parastagmatoptera flavoguttata|Regina|658[0n]
GFMAN18-091|Mantidae|Parastagmatoptera flavoguttata|Sinnamary|658[0n]
NMMAN11-0198|Mantidae|Parastagmatoptera flavoguttata|Roura|570[0n]
NMMAN11-0088|Mantidae|Parastagmatoptera flavoguttata|Roura|658[0n]
NMMAN11-0237|Mantidae|Parastagmatoptera flavoguttata|Regina|658[0n]
GFMAN18-243|Mantidae|Parastagmatoptera unipunctata|Kourou|615[0n]
GFMAN18-233|Mantidae|Parastagmatoptera unipunctata|Montsinery-Tonnegrande|658[0n]
NMMAN11-0841|Mantidae|Parastagmatoptera unipunctata|Macouria|658[0n]
GFMAN18-385|Mantidae|Parastagmatoptera unipunctata|Kourou|658[0n]
GFMAN18-232|Mantidae|Parastagmatoptera unipunctata|Iracoubo|658[0n]
GFMAN18-358|Mantidae|Parastagmatoptera unipunctata|Kourou|658[0n]
GFMAN18-357|Mantidae|Parastagmatoptera unipunctata|Kourou|658[0n]
GFMAN18-394|Mantidae|Parastagmatoptera unipunctata|Kourou|658[0n]
GFMAN18-221|Mantidae|Choeradodis strumaria|Maripasoula|580[0n]
GFMAN18-126|Mantidae|Choeradodis strumaria|Papaichton|658[0n]
GFMAN18-177|Mantidae|Choeradodis strumaria|Saul|598[0n]
GFMAN18-182|Mantidae|Choeradodis strumaria|Maripasoula|618[0n]
GFMAN18-334|Mantidae|Choeradodis rhomboidea|Maripasoula|648[1n]
NMMAN11-0190|Mantidae|Choeradodis rhomboidea|Roura|470[0n]
GFMAN18-333|Mantidae|Choeradodis rhomboidea|Roura|658[0n]
GFMAN18-413|Mantidae|Choeradodis rhomboidea|Roura|658[0n]
GFMAN18-416|Mantidae|Choeradodis rhomboidea|Roura|658[0n]
GFMAN18-023|Liturgusidae|Hagiomantis mesopoda|Roura|658[0n]
GFMAN18-282|Liturgusidae|Hagiomantis mesopoda|Regina|658[0n]
NMMAN11-0359|Liturgusidae|Liturgusa milleri|Mana|658[0n]
GFMAN18-153|Liturgusidae|Liturgusa milleri|Saul|658[0n]
GFMAN18-218|Liturgusidae|Liturgusa milleri|Maripasoula|658[0n]
GFMAN18-219|Liturgusidae|Liturgusa milleri|Maripasoula|639[0n]
NMMAN11-0917|Liturgusidae|Liturgusa milleri|Regina|658[0n]
GFMAN18-019|Liturgusidae|Liturgusa maroni|Roura|613[0n]
GFMAN18-016|Liturgusidae|Liturgusa maroni|Roura|658[0n]
NMMAN11-0925|Liturgusidae|Liturgusa maroni|Montsinery-Tonnegrande|658[0n]
NMMAN11-0930|Liturgusidae|Liturgusa maroni|Roura|658[0n]
NMMAN11-0931|Liturgusidae|Liturgusa maroni|Roura|658[0n]
GFMAN18-020|Liturgusidae|Liturgusa cayennensis|Roura|658[0n]
GFMAN18-022|Liturgusidae|Liturgusa cayennensis|Roura|658[0n]
NMMAN11-0838|Liturgusidae|Liturgusa cayennensis|Roura|658[0n]
NMMAN11-0360|Liturgusidae|Liturgusa cayennensis|Mana|658[0n]
GFMAN18-393|Liturgusidae|Liturgusa cayennensis|Kourou|562[0n]
GFMAN18-262|Acanthopidae|Acontista gracilis|Roura|658[0n]
GFMAN18-052|Acanthopidae|Acontista gracilis|Roura|658[0n]
GFMAN18-088|Acanthopidae|Acontista gracilis|Roura|658[0n]
GFMAN18-048|Acanthopidae|Acontista gracilis|Roura|658[0n]
GFMAN18-136|Acanthopidae|Acontista gracilis|Papaichton|658[0n]
NMMAN11-0187|Acanthopidae|Acontista gracilis|Regina|658[0n]
GFMAN18-062|Acanthopidae|Raptrix perspicua|Saint-Georges-de-l Oyapock|658[0n]
GFMAN18-063|Acanthopidae|Raptrix perspicua|Saint-Georges-de-l Oyapock|658[0n]
GFMAN18-064|Acanthopidae|Raptrix perspicua|Saint-Georges-de-l Oyapock|658[0n]
NMMAN11-0358|Acanthopidae|Raptrix perspicua|Mana|658[0n]
GFMAN18-044|Acanthopidae|Raptrix perspicua|Regina|658[0n]
GFMAN18-081|Acanthopidae|Raptrix perspicua|Regina|658[0n]
GFMAN18-106|Acanthopidae|Raptrix perspicua|Papaichton|658[0n]
GFMAN18-105|Acanthopidae|Raptrix perspicua|Papaichton|658[0n]
GFMAN18-115|Acanthopidae|Raptrix perspicua|Papaichton|658[0n]
GFMAN18-125|Acanthopidae|Raptrix perspicua|Papaichton|658[0n]
GFMAN18-134|Acanthopidae|Raptrix perspicua|Papaichton|658[0n]
GFMAN18-142|Acanthopidae|Raptrix perspicua|Papaichton|658[0n]
NMMAN11-0123|Acanthopidae|Raptrix perspicua|Roura|658[0n]
NMMAN11-0125|Acanthopidae|Raptrix perspicua|Roura|658[0n]
GFMAN18-174|Acanthopidae|Acontista cayennensis|Maripasoula|658[0n]
GFMAN18-135|Acanthopidae|Acontista cayennensis|Papaichton|658[0n]
GFMAN18-042|Acanthopidae|Acontista cayennensis|Matoury|658[0n]
NMMAN11-0829|Acanthopidae|Acontista cayennensis|Remire-Montjoly|658[0n]
NMMAN11-0886|Acanthopidae|Callibia diana|Roura|658[0n]
GFMAN18-304|Acanthopidae|Callibia diana|Saint-Elie|658[3n]
GFMAN18-034|Acanthopidae|Tithrone roseipennis|Maripasoula|658[0n]
GFMAN18-035|Acanthopidae|Tithrone roseipennis|Maripasoula|658[0n]
GFMAN18-194|Acanthopidae|Tithrone roseipennis|Regina|658[0n]
GFMAN18-205|Acanthopidae|Tithrone roseipennis|Sinnamary|658[0n]
GFMAN18-261|Acanthopidae|Tithrone roseipennis|Roura|658[0n]
NMMAN11-0869|Acanthopidae|Tithrone roseipennis|Roura|658[0n]
GFMAN18-151|Acanthopidae|Tithrone roseipennis|Papaichton|658[0n]
GFMAN18-412|Acanthopidae|Tithrone roseipennis|Saul|658[0n]
GFMAN18-108|Photinaidae|Cardioptera squalodon|Papaichton|658[1n]
GFMAN18-109|Photinaidae|Cardioptera squalodon|Papaichton|658[1n]
GFMAN18-110|Photinaidae|Cardioptera squalodon|Papaichton|658[1n]
GFMAN18-164|Photinaidae|Cardioptera squalodon|Maripasoula|658[1n]
NMMAN11-0908|Photinaidae|Microphotina vitripennis|Saul|658[0n]
GFMAN18-273|Photinaidae|Microphotina vitripennis|Saint-Elie|610[0n]
NMMAN11-0831|Photinaidae|Microphotina vitripennis|Saint-Elie|658[0n]
GFMAN18-301|Photinaidae|Microphotina vitripennis|Saint-Elie|658[0n]
GFMAN18-187|Photinaidae|Microphotina vitripennis|Kourou|658[0n]
GFMAN18-199|Photinaidae|Microphotina vitripennis|Kourou|658[0n]
NMMAN11-0864|Photinaidae|Microphotina vitripennis|Roura|552[1n]
GFMAN18-363|Photinaidae|Microphotina vitripennis|Sinnamary|658[0n]
GFMAN18-364|Photinaidae|Microphotina vitripennis|Sinnamary|658[0n]

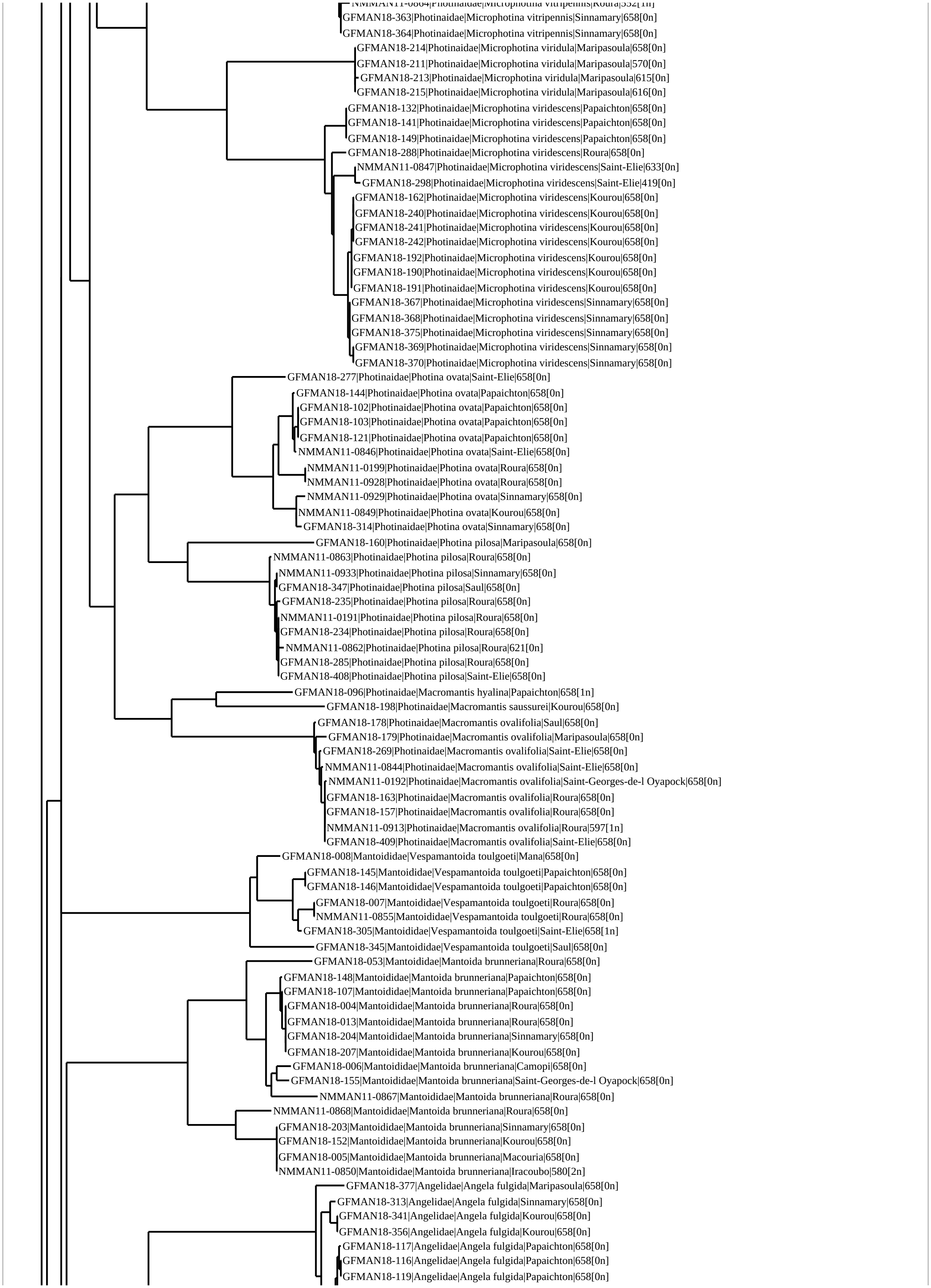
NMMAN11-0864|Photinaidae|Microphotina vitripennis|Roura|552[1n]
GFMAN18-363|Photinaidae|Microphotina vitripennis|Sinnamary|658[0n]
GFMAN18-364|Photinaidae|Microphotina vitripennis|Sinnamary|658[0n]
GFMAN18-214|Photinaidae|Microphotina viridula|Maripasoula|658[0n]
GFMAN18-211|Photinaidae|Microphotina viridula|Maripasoula|570[0n]
GFMAN18-213|Photinaidae|Microphotina viridula|Maripasoula|615[0n]
GFMAN18-215|Photinaidae|Microphotina viridula|Maripasoula|616[0n]
GFMAN18-132|Photinaidae|Microphotina viridescens|Papaichton|658[0n]
GFMAN18-141|Photinaidae|Microphotina viridescens|Papaichton|658[0n]
GFMAN18-149|Photinaidae|Microphotina viridescens|Papaichton|658[0n]
GFMAN18-288|Photinaidae|Microphotina viridescens|Roura|658[0n]
NMMAN11-0847|Photinaidae|Microphotina viridescens|Saint-Elie|633[0n]
GFMAN18-298|Photinaidae|Microphotina viridescens|Saint-Elie|419[0n]
GFMAN18-162|Photinaidae|Microphotina viridescens|Kourou|658[0n]
GFMAN18-240|Photinaidae|Microphotina viridescens|Kourou|658[0n]
GFMAN18-241|Photinaidae|Microphotina viridescens|Kourou|658[0n]
GFMAN18-242|Photinaidae|Microphotina viridescens|Kourou|658[0n]
GFMAN18-192|Photinaidae|Microphotina viridescens|Kourou|658[0n]
GFMAN18-190|Photinaidae|Microphotina viridescens|Kourou|658[0n]
GFMAN18-191|Photinaidae|Microphotina viridescens|Kourou|658[0n]
GFMAN18-367|Photinaidae|Microphotina viridescens|Sinnamary|658[0n]
GFMAN18-368|Photinaidae|Microphotina viridescens|Sinnamary|658[0n]
GFMAN18-375|Photinaidae|Microphotina viridescens|Sinnamary|658[0n]
GFMAN18-369|Photinaidae|Microphotina viridescens|Sinnamary|658[0n]
GFMAN18-370|Photinaidae|Microphotina viridescens|Sinnamary|658[0n]
GFMAN18-277|Photinaidae|Photina ovata|Saint-Elie|658[0n]
GFMAN18-144|Photinaidae|Photina ovata|Papaichton|658[0n]
GFMAN18-102|Photinaidae|Photina ovata|Papaichton|658[0n]
GFMAN18-103|Photinaidae|Photina ovata|Papaichton|658[0n]
GFMAN18-121|Photinaidae|Photina ovata|Papaichton|658[0n]
NMMAN11-0846|Photinaidae|Photina ovata|Saint-Elie|658[0n]
NMMAN11-0199|Photinaidae|Photina ovata|Roura|658[0n]
NMMAN11-0928|Photinaidae|Photina ovata|Roura|658[0n]
NMMAN11-0929|Photinaidae|Photina ovata|Sinnamary|658[0n]
NMMAN11-0849|Photinaidae|Photina ovata|Kourou|658[0n]
GFMAN18-314|Photinaidae|Photina ovata|Sinnamary|658[0n]
GFMAN18-160|Photinaidae|Photina pilosa|Maripasoula|658[0n]
NMMAN11-0863|Photinaidae|Photina pilosa|Roura|658[0n]
NMMAN11-0933|Photinaidae|Photina pilosa|Sinnamary|658[0n]
GFMAN18-347|Photinaidae|Photina pilosa|Saul|658[0n]
GFMAN18-235|Photinaidae|Photina pilosa|Roura|658[0n]
NMMAN11-0191|Photinaidae|Photina pilosa|Roura|658[0n]
GFMAN18-234|Photinaidae|Photina pilosa|Roura|658[0n]
NMMAN11-0862|Photinaidae|Photina pilosa|Roura|621[0n]
GFMAN18-285|Photinaidae|Photina pilosa|Roura|658[0n]
GFMAN18-408|Photinaidae|Photina pilosa|Saint-Elie|658[0n]
GFMAN18-096|Photinaidae|Macromantis hyalina|Papaichton|658[1n]
GFMAN18-198|Photinaidae|Macromantis saussurei|Kourou|658[0n]
GFMAN18-178|Photinaidae|Macromantis ovalifolia|Saul|658[0n]
GFMAN18-179|Photinaidae|Macromantis ovalifolia|Maripasoula|658[0n]
GFMAN18-269|Photinaidae|Macromantis ovalifolia|Saint-Elie|658[0n]
NMMAN11-0844|Photinaidae|Macromantis ovalifolia|Saint-Elie|658[0n]
NMMAN11-0192|Photinaidae|Macromantis ovalifolia|Saint-Georges-de-l Oyapock|658[0n]
GFMAN18-163|Photinaidae|Macromantis ovalifolia|Roura|658[0n]
GFMAN18-157|Photinaidae|Macromantis ovalifolia|Roura|658[0n]
NMMAN11-0913|Photinaidae|Macromantis ovalifolia|Roura|597[1n]
GFMAN18-409|Photinaidae|Macromantis ovalifolia|Saint-Elie|658[0n]
GFMAN18-008|Mantoididae|Vespamantoida toulgoeti|Mana|658[0n]
GFMAN18-145|Mantoididae|Vespamantoida toulgoeti|Papaichton|658[0n]
GFMAN18-146|Mantoididae|Vespamantoida toulgoeti|Papaichton|658[0n]
GFMAN18-007|Mantoididae|Vespamantoida toulgoeti|Roura|658[0n]
NMMAN11-0855|Mantoididae|Vespamantoida toulgoeti|Roura|658[0n]
GFMAN18-305|Mantoididae|Vespamantoida toulgoeti|Saint-Elie|658[1n]
GFMAN18-345|Mantoididae|Vespamantoida toulgoeti|Saul|658[0n]
GFMAN18-053|Mantoididae|Mantoida brunneriana|Roura|658[0n]
GFMAN18-148|Mantoididae|Mantoida brunneriana|Papaichton|658[0n]
GFMAN18-107|Mantoididae|Mantoida brunneriana|Papaichton|658[0n]
GFMAN18-004|Mantoididae|Mantoida brunneriana|Roura|658[0n]
GFMAN18-013|Mantoididae|Mantoida brunneriana|Roura|658[0n]
GFMAN18-204|Mantoididae|Mantoida brunneriana|Sinnamary|658[0n]
GFMAN18-207|Mantoididae|Mantoida brunneriana|Kourou|658[0n]
GFMAN18-006|Mantoididae|Mantoida brunneriana|Camopi|658[0n]
GFMAN18-155|Mantoididae|Mantoida brunneriana|Saint-Georges-de-l Oyapock|658[0n]
NMMAN11-0867|Mantoididae|Mantoida brunneriana|Roura|658[0n]
NMMAN11-0868|Mantoididae|Mantoida brunneriana|Roura|658[0n]
GFMAN18-203|Mantoididae|Mantoida brunneriana|Sinnamary|658[0n]
GFMAN18-152|Mantoididae|Mantoida brunneriana|Kourou|658[0n]
GFMAN18-005|Mantoididae|Mantoida brunneriana|Macouria|658[0n]
NMMAN11-0850|Mantoididae|Mantoida brunneriana|Iracoubo|580[2n]
GFMAN18-377|Angelidae|Angela fulgida|Maripasoula|658[0n]
GFMAN18-313|Angelidae|Angela fulgida|Sinnamary|658[0n]
GFMAN18-341|Angelidae|Angela fulgida|Kourou|658[0n]
GFMAN18-356|Angelidae|Angela fulgida|Kourou|658[0n]
GFMAN18-117|Angelidae|Angela fulgida|Papaichton|658[0n]
GFMAN18-116|Angelidae|Angela fulgida|Papaichton|658[0n]
GFMAN18-119|Angelidae|Angela fulgida|Papaichton|658[0n]

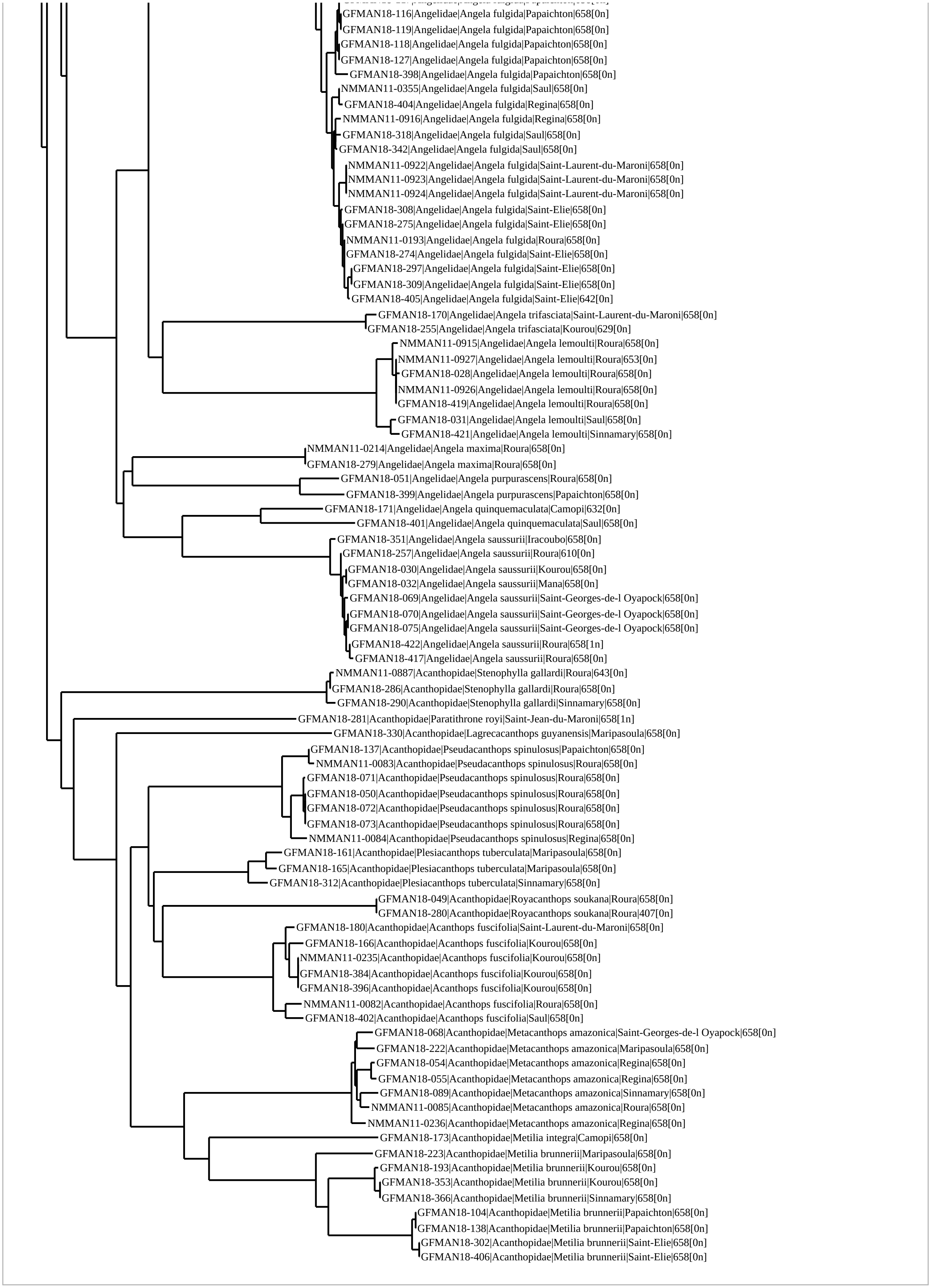
GFMAN18-116|Angelidae|Angela fulgida|Papaichton|658[0n]
GFMAN18-119|Angelidae|Angela fulgida|Papaichton|658[0n]
GFMAN18-118|Angelidae|Angela fulgida|Papaichton|658[0n]
GFMAN18-127|Angelidae|Angela fulgida|Papaichton|658[0n]
GFMAN18-398|Angelidae|Angela fulgida|Papaichton|658[0n]
NMMAN11-0355|Angelidae|Angela fulgida|Saul|658[0n]
GFMAN18-404|Angelidae|Angela fulgida|Regina|658[0n]
NMMAN11-0916|Angelidae|Angela fulgida|Regina|658[0n]
GFMAN18-318|Angelidae|Angela fulgida|Saul|658[0n]
GFMAN18-342|Angelidae|Angela fulgida|Saul|658[0n]
NMMAN11-0922|Angelidae|Angela fulgida|Saint-Laurent-du-Maroni|658[0n]
NMMAN11-0923|Angelidae|Angela fulgida|Saint-Laurent-du-Maroni|658[0n]
NMMAN11-0924|Angelidae|Angela fulgida|Saint-Laurent-du-Maroni|658[0n]
GFMAN18-308|Angelidae|Angela fulgida|Saint-Elie|658[0n]
GFMAN18-275|Angelidae|Angela fulgida|Saint-Elie|658[0n]
NMMAN11-0193|Angelidae|Angela fulgida|Roura|658[0n]
GFMAN18-274|Angelidae|Angela fulgida|Saint-Elie|658[0n]
GFMAN18-297|Angelidae|Angela fulgida|Saint-Elie|658[0n]
GFMAN18-309|Angelidae|Angela fulgida|Saint-Elie|658[0n]
GFMAN18-405|Angelidae|Angela fulgida|Saint-Elie|642[0n]
GFMAN18-170|Angelidae|Angela trifasciata|Saint-Laurent-du-Maroni|658[0n]
GFMAN18-255|Angelidae|Angela trifasciata|Kourou|629[0n]
NMMAN11-0915|Angelidae|Angela lemoulti|Roura|658[0n]
NMMAN11-0927|Angelidae|Angela lemoulti|Roura|653[0n]
GFMAN18-028|Angelidae|Angela lemoulti|Roura|658[0n]
NMMAN11-0926|Angelidae|Angela lemoulti|Roura|658[0n]
GFMAN18-419|Angelidae|Angela lemoulti|Roura|658[0n]
GFMAN18-031|Angelidae|Angela lemoulti|Saul|658[0n]
GFMAN18-421|Angelidae|Angela lemoulti|Sinnamary|658[0n]
NMMAN11-0214|Angelidae|Angela maxima|Roura|658[0n]
GFMAN18-279|Angelidae|Angela maxima|Roura|658[0n]
GFMAN18-051|Angelidae|Angela purpurascens|Roura|658[0n]
GFMAN18-399|Angelidae|Angela purpurascens|Papaichton|658[0n]
GFMAN18-171|Angelidae|Angela quinquemaculata|Camopi|632[0n]
GFMAN18-401|Angelidae|Angela quinquemaculata|Saul|658[0n]
GFMAN18-351|Angelidae|Angela saussurii|Iracoubo|658[0n]
GFMAN18-257|Angelidae|Angela saussurii|Roura|610[0n]
GFMAN18-030|Angelidae|Angela saussurii|Kourou|658[0n]
GFMAN18-032|Angelidae|Angela saussurii|Mana|658[0n]
GFMAN18-069|Angelidae|Angela saussurii|Saint-Georges-de-l Oyapock|658[0n]
GFMAN18-070|Angelidae|Angela saussurii|Saint-Georges-de-l Oyapock|658[0n]
GFMAN18-075|Angelidae|Angela saussurii|Saint-Georges-de-l Oyapock|658[0n]
GFMAN18-422|Angelidae|Angela saussurii|Roura|658[1n]
GFMAN18-417|Angelidae|Angela saussurii|Roura|658[0n]
NMMAN11-0887|Acanthopidae|Stenophylla gallardi|Roura|643[0n]
GFMAN18-286|Acanthopidae|Stenophylla gallardi|Roura|658[0n]
GFMAN18-290|Acanthopidae|Stenophylla gallardi|Sinnamary|658[0n]
GFMAN18-281|Acanthopidae|Paratithrone royi|Saint-Jean-du-Maroni|658[1n]
GFMAN18-330|Acanthopidae|Lagrecacanthops guyanensis|Maripasoula|658[0n]
GFMAN18-137|Acanthopidae|Pseudacanthops spinulosus|Papaichton|658[0n]
NMMAN11-0083|Acanthopidae|Pseudacanthops spinulosus|Roura|658[0n]
GFMAN18-071|Acanthopidae|Pseudacanthops spinulosus|Roura|658[0n]
GFMAN18-050|Acanthopidae|Pseudacanthops spinulosus|Roura|658[0n]
GFMAN18-072|Acanthopidae|Pseudacanthops spinulosus|Roura|658[0n]
GFMAN18-073|Acanthopidae|Pseudacanthops spinulosus|Roura|658[0n]
NMMAN11-0084|Acanthopidae|Pseudacanthops spinulosus|Regina|658[0n]
GFMAN18-161|Acanthopidae|Plesiacanthops tuberculata|Maripasoula|658[0n]
GFMAN18-165|Acanthopidae|Plesiacanthops tuberculata|Maripasoula|658[0n]
GFMAN18-312|Acanthopidae|Plesiacanthops tuberculata|Sinnamary|658[0n]
GFMAN18-049|Acanthopidae|Royacanthops soukana|Roura|658[0n]
GFMAN18-280|Acanthopidae|Royacanthops soukana|Roura|407[0n]
GFMAN18-180|Acanthopidae|Acanthops fuscifolia|Saint-Laurent-du-Maroni|658[0n]
GFMAN18-166|Acanthopidae|Acanthops fuscifolia|Kourou|658[0n]
NMMAN11-0235|Acanthopidae|Acanthops fuscifolia|Kourou|658[0n]
GFMAN18-384|Acanthopidae|Acanthops fuscifolia|Kourou|658[0n]
GFMAN18-396|Acanthopidae|Acanthops fuscifolia|Kourou|658[0n]
NMMAN11-0082|Acanthopidae|Acanthops fuscifolia|Roura|658[0n]
GFMAN18-402|Acanthopidae|Acanthops fuscifolia|Saul|658[0n]
GFMAN18-068|Acanthopidae|Metacanthops amazonica|Saint-Georges-de-l Oyapock|658[0n]
GFMAN18-222|Acanthopidae|Metacanthops amazonica|Maripasoula|658[0n]
GFMAN18-054|Acanthopidae|Metacanthops amazonica|Regina|658[0n]
GFMAN18-055|Acanthopidae|Metacanthops amazonica|Regina|658[0n]
GFMAN18-089|Acanthopidae|Metacanthops amazonica|Sinnamary|658[0n]
NMMAN11-0085|Acanthopidae|Metacanthops amazonica|Roura|658[0n]
NMMAN11-0236|Acanthopidae|Metacanthops amazonica|Regina|658[0n]
GFMAN18-173|Acanthopidae|Metilia integra|Camopi|658[0n]
GFMAN18-223|Acanthopidae|Metilia brunnerii|Maripasoula|658[0n]
GFMAN18-193|Acanthopidae|Metilia brunnerii|Kourou|658[0n]
GFMAN18-353|Acanthopidae|Metilia brunnerii|Kourou|658[0n]
GFMAN18-366|Acanthopidae|Metilia brunnerii|Sinnamary|658[0n]
GFMAN18-104|Acanthopidae|Metilia brunnerii|Papaichton|658[0n]
GFMAN18-138|Acanthopidae|Metilia brunnerii|Papaichton|658[0n]
GFMAN18-302|Acanthopidae|Metilia brunnerii|Saint-Elie|658[0n]
GFMAN18-406|Acanthopidae|Metilia brunnerii|Saint-Elie|658[0n]
